# The fossil record and taphonomy of butterflies and moths (Insecta, Lepidoptera): implications for evolutionary diversity and divergence-time estimates

**DOI:** 10.1186/s12862-015-0290-8

**Published:** 2015-02-04

**Authors:** Jae-Cheon Sohn, Conrad C Labandeira, Donald R Davis

**Affiliations:** Department of Entomology, Smithsonian Institution, National Museum of Natural History, Washington, DC USA; Department of Paleobiology, Smithsonian Institution, National Museum of Natural History, Washington, DC USA; Department of Entomology, University of Maryland, College Park, MD USA; College of Life Sciences, Capital Normal University, Beijing, China

**Keywords:** Lepidoptera, Fossil record, Taphonomy, Diversity, Divergence-time estimation

## Abstract

**Background:**

It is conventionally accepted that the lepidopteran fossil record is significantly incomplete when compared to the fossil records of other, very diverse, extant insect orders. Such an assumption, however, has been based on cumulative diversity data rather than using alternative statistical approaches from actual specimen counts.

**Results:**

We reviewed documented specimens of the lepidopteran fossil record, currently consisting of 4,593 known specimens that are comprised of 4,262 body fossils and 331 trace fossils. The temporal distribution of the lepidopteran fossil record shows significant bias towards the late Paleocene to middle Eocene time interval. Lepidopteran fossils also record major shifts in preservational style and number of represented localities at the Mesozoic stage and Cenozoic epoch level of temporal resolution. Only 985 of the total known fossil specimens (21.4%) were assigned to 23 of the 40 extant lepidopteran superfamilies. Absolute numbers and proportions of preservation types for identified fossils varied significantly across superfamilies. The secular increase of lepidopteran family-level diversity through geologic time significantly deviates from the general pattern of other hyperdiverse, ordinal-level lineages.

**Conclusion:**

Our statistical analyses of the lepidopteran fossil record show extreme biases in preservation type, age, and taxonomic composition. We highlight the scarcity of identified lepidopteran fossils and provide a correspondence between the latest lepidopteran divergence-time estimates and relevant fossil occurrences at the superfamily level. These findings provide caution in interpreting the lepidopteran fossil record through the modeling of evolutionary diversification and in determination of divergence time estimates.

**Electronic supplementary material:**

The online version of this article (doi:10.1186/s12862-015-0290-8) contains supplementary material, which is available to authorized users.

## Background

The Lepidoptera, including moths, butterflies and skippers, are one of the most speciose lineages on the Earth, currently consisting of over 160,000 described species and possibly approximating a half million total species [1]. The elevated species diversity of Lepidoptera represents nearly 3% of the extant world biota [2,3]. Lepidoptera play fundamental roles in terrestrial ecosystems, principally through larvae as herbivores and adults as pollinators [4,5], and at higher trophic levels Lepidoptera serve as an important food source for other animals [6]. On an aesthetic note, many butterflies provide an important source of wonder and beauty [7], and perhaps as a result, are one of the most extensively studied of invertebrate groups. In spite of their importance in global biodiversity, the evolutionary history of Lepidoptera is poorly known. This mostly is attributable to their poor fossil record that contrasts to other, much better represented, major insect orders [8-11]. From this paucity of fossils, evolutionary hypotheses for Lepidoptera were largely based on the extant fauna [1,12,13], although there were subsequent attempts to use fossils in estimating their divergence-time dates [14-16]. Robust molecular dating requires multiple, reliably-identified fossils, each fossil of which is sufficiently old to address a relevant divergence event in a deep-time phylogeny [17,18]. These requirements often are difficult to meet for the depauperate lepidopteran fossil record. Moreover, there are major concerns of the lepidopteran fossil record that involve taxonomic and geochronologic biases as well as the reliability of fossil identifications [11,19]. These data biases and identification issues in the lepidopteran fossil record have not been thoroughly explored.

The earliest fossil reliably identified as a member of the Lepidoptera is *Archaeolepis mane* Whalley, from a Lower Jurassic calcareous flatstone deposit in England [20,21]. Other early lepidopteran fossils are known from the Middle Jurassic [22-24], reviewed preliminarily by Skalski [25] and Kristensen and Skalski [10]. However, the greatest amount of material originates from mid Cenozoic compression–impression and amber–copal deposits [21,26]. Prevailing views regarding such a geochronological bias resulted in the notion that the Lepidoptera, of all insect orders, evolved most recently [21,27-29], and in particular diversified, perhaps in concert, with angiosperms [13,15,30,31]. These proposals, nevertheless, have been based on anecdotal rather than systematic approaches to the lepidopteran fossil record which recently became feasible from a comprehensive compilation of documented fossil Lepidoptera [32] (also see [33]).

The goal of this paper is to provide an overview of the lepidopteran fossil record based on data from Sohn et al. [32] and Sohn and Lamas [33], including statistical summaries of preservational categories, age distributions, and taxonomic composition. The biases and other issues originating from these data are discussed for identifying aspects of the lepidopteran fossil record that need to be addressed by future molecular dating analyses. The resulting patterns are compared with previous views, allowing an updated revision of lepidopteran evolution.

## Methods

### Data collection

Sohn et al. [32] provide a comprehensive compilation of known lepidopteran fossils. Their catalog includes 4,593 fossil specimens reliably assigned to Lepidoptera. The total number was based on a conservative, cumulative tabulation of fossil taxonomic entries such that ambiguous reports were kept to a minimum. For example, taxonomic accounts listing multiple specimens were counted as two specimens, and the absence of a statement specifying the number of taxa was counted as one specimen. When body and trace fossils rarely occurred together in the same matrix, for example a psychid larva within its own case, they were counted as two specimens. This separation is attributable to the standard paleoentomological practice of describing body and trace fossils as separate taxa [34].

The resulting data were categorized by preservational type, geochronologic age and taxonomic affinities, as defined in Sohn et al. [32]. Preservational types are described, with modification, by the eight categories of Sohn et al. [32]. They are: (1), amber and copal combined into a single amber–copal category; (2), asphaltum and tar sands; (3), compression and impression fossils; (4); gut contents and coprolites of insectivorous animals; (5), peat and lignite; (6), salt deposits; (7), sieved residues; and (8), silica or other types of permineralization [35]. Each preservational type for the 4,593 fossil occurrences [32] were subdivided into two categories, body and trace fossils. A body fossil is defined as consisting of the entire or partial body (frequently wings) of a lepidopteran egg, larva, pupa or adult in sedimentary matrix. By contrast, a trace fossil consists of plant damage, a teratology or otherwise herbivore activity caused by a lepidopteran, consisting principally of leaf-mines, other feeding marks, or any product derived from lepidopteran activity, such as larval domicile cases and more rarely, associated frass. Body and trace fossils ambiguously affiliated with Lepidoptera were excluded.

**Figure 1:**
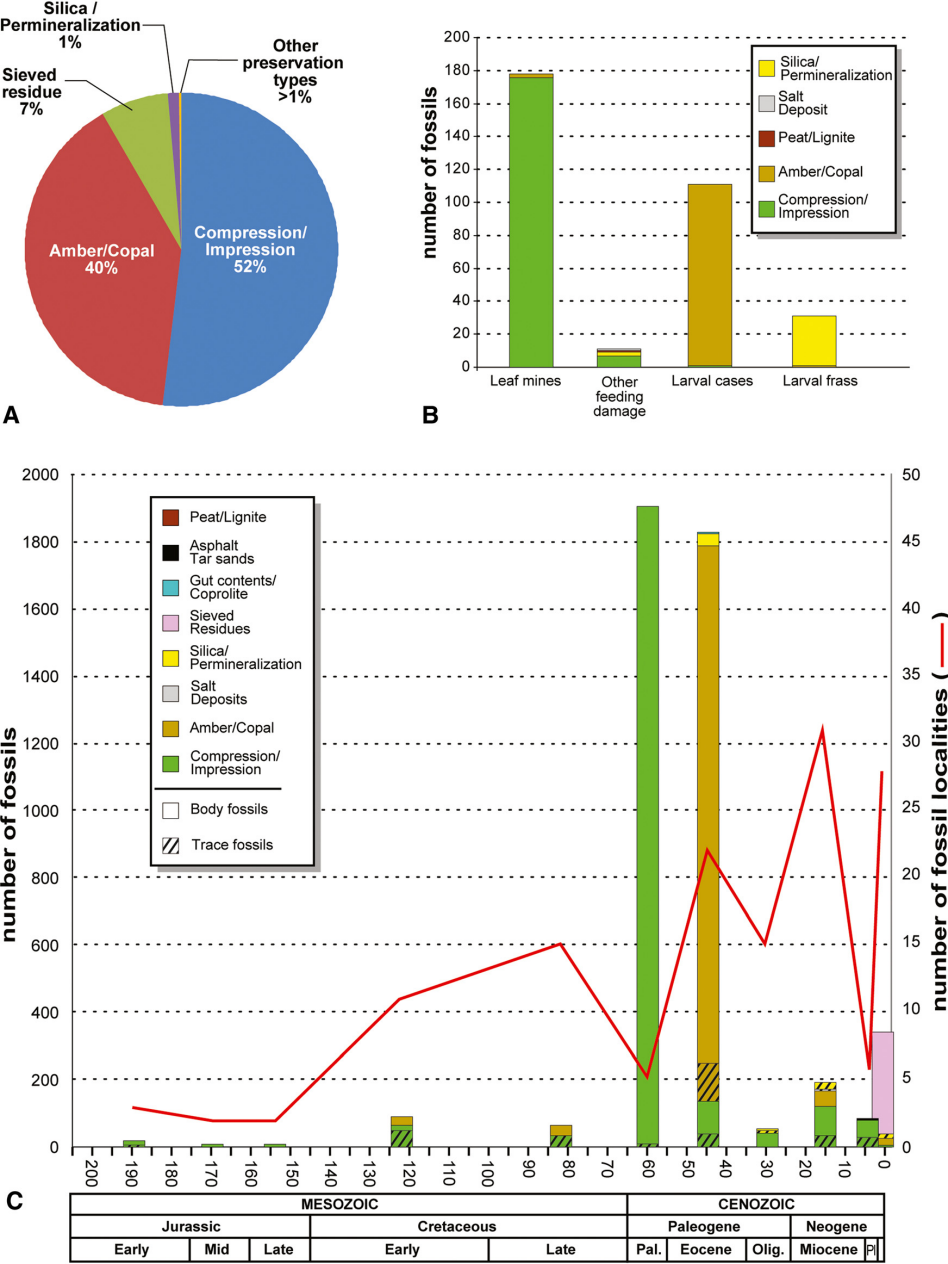
**Proportional representation of 4,593 lepidopteran fossils categorized by preservational type, abundance, age, and associated locality, documented in Sohn et al. [** 32 **]. (A)**, Proportional representation of preservational types in the lepidopteran body-fossil record (N = 4,262). **(B)**, Proportional representation of trace-fossil types in the lepidopteran fossil record (N = 331). **(C)**, Frequency distribution of lepidopteran body and trace fossils (N = 4,561) by geochronologic age, preservational type, abundance, and number (N = 145) of lepidopteran-bearing localities. The age of Baltic Amber is taken as middle Eocene, discussed in Labandeira [74], and the overall geochronology is after Gradstein et al. [36], indicated by the scale bar at bottom, in millions of years. Abbreviations: Mid, Middle; Pal, Paleocene; Olig., Oligocene; Pl, Pliocene; the Pleistocene + Holocene occurs to the right of the Pliocene.

The 4,561 body- and trace fossils of known geological age were divided into temporally delimited bins at the epoch or stage intervals of resolution, and further subdivided by their preservation type. Age determinations are provided in Sohn et al. [32] and Sohn and Lamas [33]. Each fossil age date was given at the midpoint of an epoch or stage interval, which also was chosen for graphical representation. The geologic time scale of Gradstein et al. [36], the international standard, was used. We combined fossil and subfossil occurrences of Pleistocene and Holocene age into a single time interval in Figure 1C (each data point is available in Table 1). The number of fossil deposits at each age interval was calculated based on data from the primary geological and paleoentomological literature. Because of the encompassing spatiotemporal scale involved in plotting the data, multiple occurrences of similarly dated lepidopteran fossils, within about five million years of each other, were combined into a single, composite data point, indicated in Additional file 1.

**Table 1:** **The number of trace and body fossils by their preservational type and age**

	**Trace fossils**					**Body fossils**						
**Time intervals**	**CI**	**AM**	**SI**	**SA**	**PE**	**CI**	**AM**	**SI**	**SR**	**GC**	**AS**	**PE**
**Early Jurassic**	1	0	0	0	0	14	0	0	0	0	0	0
**Middle Jurassic**	0	0	0	0	0	5	0	0	0	0	0	0
**Late Jurassic**	0	0	0	0	0	6	0	0	0	0	0	0
**Early Cretaceous**	47	0	0	0	0	15	26	0	0	0	0	0
**Late Cretaceous**	29	0	0	0	0	1	31	1	1	0	0	0
**Early Paleocene**	4	0	0	0	0	1	0	0	0	0	0	0
**Middle Paleocene**	0	0	0	0	0	1	0	0	0	0	0	0
**Late Paleocene**	3	0	0	0	0	1892	0	0	0	0	0	0
**Early Eocene**	4	0	0	0	0	12	0	0	0	0	0	0
**Middle Eocene**	28	111	0	0	0	18	1532	37	0	4	0	0
**Late Eocene**	6	0	0	0	0	67	5	0	0	0	0	0
**Early Oligocene**	0	0	0	0	0	27	1	1	0	0	0	0
**Late Oligocene**	2	0	4	0	0	12	0	2	0	0	0	0
**Early Miocene**	9	2	0	0	0	61	44	2	0	0	0	0
**Middle Miocene**	15	0	0	1	0	14	1	0	0	0	0	0
**Late Miocene**	7	0	20	0	0	14	0	0	0	0	0	0
**Early Pliocene**	2	0	0	0	0	0	0	0	0	0	0	0
**Late Pliocene**	24	0	0	0	0	52	0	1	0	0	1	0
**Pleistocene**	0	0	8	0	1	2	18	0	296	0	1	1
**Holocene**	0	0	0	0	0	3	4	2	4	0	0	0

Columns other than time intervals indicate preservation types: AM, amber and copal; AS, asphaltum and tar sands; CI, compressions and impressions; GC, gut contents and coprolites; PE, peat and lignite; SA, salt deposits; SR, sieved residues; and SI, silica and other forms of permineralization.

**Figure 2:**
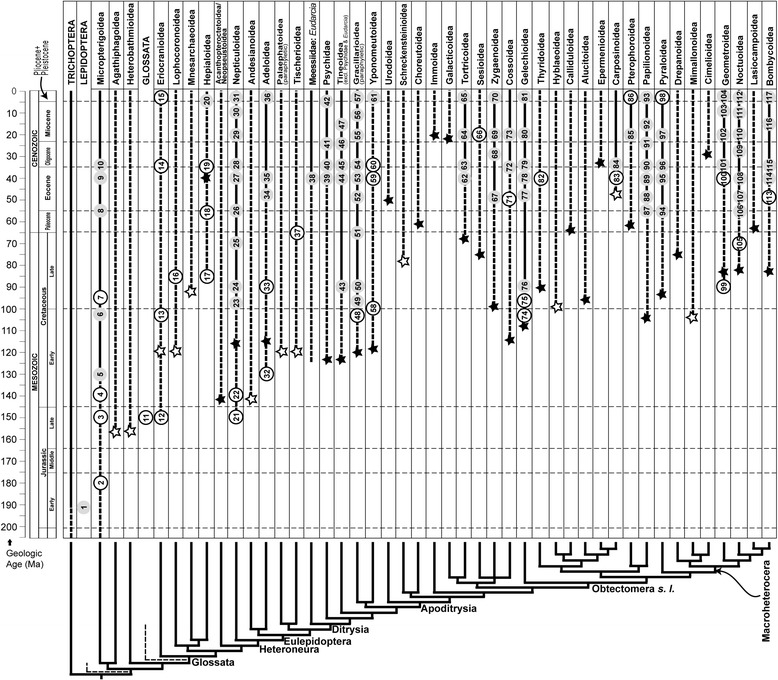
**Fossil records of lepidopteran superfamilies arranged by recent molecular phylogenetic studies.** Circles on vertical lines indicate important fossil occurrences, representing from one occurrence to a temporally constrained cluster of multiple occurrences present within an approximate 5 million-year interval. White circles indicate putative fossil identifications; gray circles indicate the fossil identifications based on reasonable evidence. The solid vertical lines spanning geologic time indicate definitive fossil evidence, whereas dashed line segments represent no or unreliable fossil evidence. The numbers within the circles were assigned successively along each lineage from lower left to upper right of the cladogram; see Additional file 1 for details. The “stars” indicate the divergence time estimates by Wahlberg et al. [15]: crown group (solid stars) or stem group ages (open stars). The cladogram and higher-group labels in left column follow Regier et al. [40] with a few modifications for topologically unstable superfamilies. A few minor superfamilies such as Douglasioidea, Simaethistoidea, and Whalleyanoidea were omitted. The age of Baltic Amber is taken as middle Eocene, discussed in Labandeira [74], the overall geochronology is after Gradstein et al. [36]. Abbreviation: Ma, millions of years.

The taxonomic affinities of fossil occurrences were tabulated and assigned to lepidopteran superfamilies, following the assignments of Sohn et al. [32] and Sohn and Lamas [33]. Lepidopteran classification follows the system of van Nieukerken et al. [37]. It is important to note that our tabulations for superfamily composition (Table 2) did not distinguish between securely identified fossils (gray data points in Figure 2), from those whose taxonomic assignment was more questionable (white data points in Figure 2). The total number of fossils for each superfamily was partitioned into their respective preservational types (Table 2).

**Table 2:** **Taxonomic and preservational modes of lepidopteran fossils**

**Superfamily**	**CI**	**CI&T**	**AM**	**AM&T**	**CO**	**SI**	**SI&T**	**SR**	**GC**	**AS**	**PET**	**Total**
**Tineoidea**	3	0	105	96	0	8	1	1	0	0	0	214
**Papilionoidea**	81	0	50	0	9	0	0	1	1	0	0	142
**Noctuoidea**	30	0	3	1	6	2	0	67	0	1	0	110
**Nepticuloidea**	2	97	2	0	2	0	0	0	0	0	0	103
**Gelechioidea**	3	14	76	0	0	0	0	0	0	0	0	93
**Tortricoidea**	2	0	78	0	1	0	0	0	0	0	1	82
**Bombycoidea**	9	0	1	0	0	40	0	1	1	1	0	53
**Gracillarioidea**	1	39	4	1	0	0	0	0	0	0	0	45
**Micropterigoidea**	10	0	21	0	0	0	0	0	1	0	0	32
**Yponomeutoidea**	4	3	14	1	1	0	0	0	0	0	0	23
**Adeloidea**	0	8	12	0	0	0	0	0	0	0	0	20
**Geometroidea**	8	0	3	0	4	0	0	2	0	0	0	17
**Pyraloidea**	8	0	2	0	1	0	0	0	0	0	0	11
**Zygaenoidea**	9	0	0	0	0	0	0	0	0	1	0	10
**Hepialoidea**	7	0	0	0	0	2	0	0	0	0	0	9
**Cossoidea**	5	0	1	0	0	0	0	0	1	0	0	7
**Eriocranioidea**	2	1	2	0	0	0	0	0	0	0	0	5
**Pterophoroidea**	3	0	0	0	0	0	0	0	0	0	0	3
**Carposinoidea**	1	0	1	0	0	0	0	0	0	0	0	2
**Lophocoronoidea**	0	0	1	0	0	0	0	0	0	0	0	1
**Mnesarchaeoidea**	0	0	1	0	0	0	0	0	0	0	0	1
**Tischerioidea**	0	1	0	0	0	0	0	0	0	0	0	1
**Thyridoidea**	0	0	1	0	0	0	0	0	0	0	0	1

The numbers of the fossil specimens are shown in each column. The lepidopteran superfamilies are arranged by numerical rank order of total fossil specimens. Preservational mode abbreviations: AM, amber and copal; AS, asphaltum and tar sands; CI, compressions and impressions; GC, gut contents and coprolites; PE, peat and lignite; SA, salt deposits; SR, sieved residues; and SI, silica and other forms of permineralization.

### Geochronological occurrence of lepidopteran fossils

Lepidopteran fossils were sorted by superfamilies and their geochronologic durations were plotted through geologic time. The interrelationships among these superfamilies follow the results of recent molecular phylogenetic studies [38-41]. Weakly supported clades were collapsed into polytomies. Divergence times of superfamily-level lineages follow Wahlberg et al. [15]. Of the published estimates of Bayesian posterior probabilities, the age of a crown group was primarily chosen, unless the lineage was based on a single species. *Eudarcia* (Meessiidae) was not sampled in Wahlberg et al. [15], and thus its divergence time was adjusted by its sister-group age. Fossil occurrences from which the data are derived are tabulated in Additional file 1. We recognize an approximate and somewhat subjective distinction between more and less reliable identifications. Less reliable fossils involve morphotypes with uncertain taxonomic affiliation, such as trace-fossil affiliations identified with extant presumptive descendants, or body fossils whose original affiliations have been questioned subsequently in the literature. It needs to be noted that we take the literature at face value; our role is not to verify or correct fossil identifications.

### Estimation of family-level diversity

**Figure 3:**
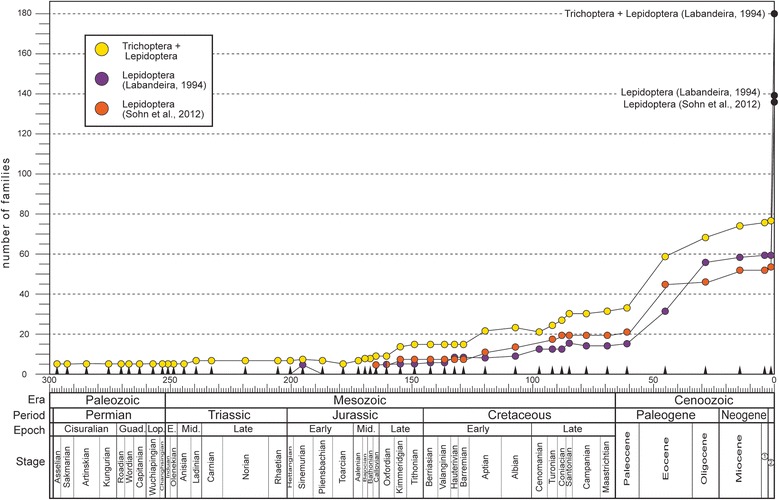
**Family-level diversity of the Lepidoptera and Amphiesmenoptera (Lepidoptera + Trichoptera).** Modern data for the Amphiesmenoptera is from Labandeira [44], shown as yellow circles; a mid 1990’s understanding of Lepidopteran history is from Labandeira [44], as purple circles; and current understanding of lepidopteran history is from Sohn et al. [32], as orange circles. The range-through method tabulating occurrence data was used, with data plotted at interval midpoints [9]. The age of Baltic Amber is taken as middle Eocene, discussed in Labandeira [74]; the geochronology at bottom is after Gradstein et al. [36]. The scale bar at bottom designates geologic time, in millions of years. Abbreviations: 1, Pliocene; 2, Pliocene + Pleistocene + Holocene.

**Figure 4:**
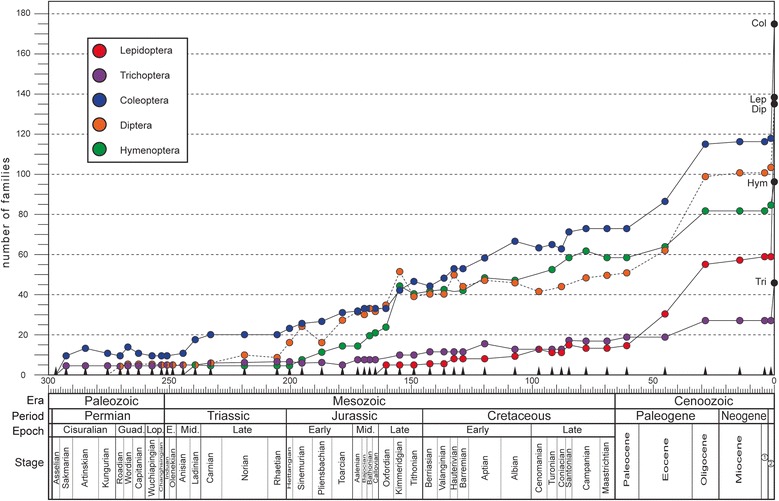
**Family-level diversity of four major, ordinal-level, holometabolous lineages.** Symbols for ordinal-level lineages: Trichoptera, purple; Coleoptera, blue; Diptera, brown; and Hymenoptera, green. All data were sourced from Labandeira [44], with updates. The range-through method was used, with data plotted at interval midpoints [9]. The age of Baltic Amber is taken as middle Eocene, discussed in Labandeira [74]; the geochronology at bottom is after Gradstein et al. [36]. The scale bar designates geologic time, in millions of years. Abbreviations: 1, Pliocene; and 2, Pleistocene + Holocene.

It has been known for some time that insect diversity analyses at the family level are suitable for inferring fossil diversity studies at other levels [42], a procedure that parallels methods such as the higher taxon approach [43], used for estimating diversity in modern ecosystems. From the fossil data, we assessed the earliest occurrences of lepidopteran families through geologic time. The raw data initially used by Labandeira and Sepkoski [9] were based on a compilation [44] with supplemental updates (Figure 3). Recently, Nicholson et al. [45] and Rainford et al. [46] provided an updated view on the evolution of insect diversity at the family level, but we retained Labandeira and Sepkoski [9] as our primary data source, and endeavored to provide updates from the more recent literature [44-46]. We also calculated lepidopteran family-level diversity data using the latest compendium of lepidopteran fossils [32], which was compared to amphiesmenopteran (Trichoptera + Lepidoptera) diversity (Figure 3). This contrast indicates that from about two-thirds to three-fourths of amphiesmenopteran diversity throughout most of the Cretaceous and Cenozoic has consisted of lepidopteran diversity. Another useful comparison of the lepidopteran fossil record was consideration of only those holometabolous orders with comparably elevated extant diversity, such as Coleoptera, Diptera and Hymenoptera (Figure 4), and the closely related Trichoptera of the Amphiesmenoptera, consisting of Lepidoptera + Trichoptera (Figure 3). The range-through method [9] was used for tracking the family-level diversity of the insect groups over geologic time. The number of families for each interval was tabulated at the midpoints of geological stage or epoch intervals. These data were statistically analyzed using Microsoft® Office Excel 2010. Linear and exponential regressions were chosen to model the relationship among variables.

## Results

### Taphonomic trends

We assess the influence of taphonomy and taxonomic affiliation on the lepidopteran fossil record. Our analyses of 4,593 specimens assigned to the Lepidoptera was sourced from the latest catalog of fossil and subfossil specimens [32], including updated corrections [33]. Of the 4,593 specimens in the database, 985 (21.4%) were assigned to a superfamily, based on identifications of fossil specimens from the primary literature or in subsequent reviews. Only 328 of these fossil specimens belonged to superfamilies that are known to occur in the fossil record, based on 236 described, fossil lepidopteran species. Of the total number of specimens, 4,262 (92.8%) were body fossils and 331 (7.2%) specimens were trace fossils. When the body-fossil fraction of 4,262 specimens were sorted by preservational type, 52.0% (2,218) were compression–impression fossils and 40.0% (1,646) were inclusions in amber and copal; both preservational modes represented 92.0% of all lepidopteran body fossils (Figure 1A). Of the remaining body fossils, 7.0% (298) were sieved residues, representing mostly specimens from Pliocene–Pleistocene glacial deposits. All other types of preservation consisted of asphaltum and tar sands, gut contents and coprolites, peat and lignites, salt deposits, and silica and other types of permineralization, which collectively accounted for somewhat less than 1% (100) of body-fossil preservational types (Figure 1A).

Within trace fossils, preservational types consisted principally of compression–impression fossils, representing 55.6% (184) of the total, whereas amber–copal inclusions contributed 34.1% (113), both of which accounted for 89.7% of all specimens (Figure 1B). In addition, the most frequent occurrence of trace fossils was leaf mines, representing 57.1% (178), followed by larval cases (33.5%, 111), and larval frass (9.4%, 31) (Figure 1B). Leaf-mines were predominantly preserved as compressions or impressions (55.0%, 176), whereas larval cases and frass were recovered almost exclusively from amber (34.4%, 110); silica and other forms of permineralization constituted a subordinate preservational type (9.4%, 30). All other preservational types were minor, representing 1.2% (4) of the total (Figure 1B).

The 4,561 lepidopteran fossils whose age is known spanned a time interval ranging from the Early Jurassic to the Holocene, or ca. 195 million years. During this interval there are two elevated frequency peaks in their distribution (Figure 1C). One elevated mode of 1,901 specimens is in the Paleocene, and the other subequal mode of 1,824 specimens occurs during the Eocene. A minor peak of 340 specimens is present in the Pleistocene to Holocene. Other than these three peaks, the number of recovered lepidopteran fossils consistently was less than 120 specimens. The composition of preservational types significantly varied among geologic epochs, seven of which (Early Jurassic, Middle Jurassic, Late Jurassic, late Paleocene, Oligocene, middle Miocene, Pliocene) consisted predominantly or almost entirely of compression–impression body fossils (Figure 1C; Table 1). Middle and late Eocene fossils (n = 1,730) overwhelmingly consisted of body inclusions in amber and Pliocene + Pleistocene deposits overwhelmingly were composed of sieved residues (Figure 1C; Table 1).

Lepidopteran fossils have been found from 145 localities worldwide. From a sort of the localities by geologic age, the greatest numbers, in decreasing rank order, were the (1) early Miocene (31 localities), (2) Pleistocene + Holocene (23 localities), (3), middle and late Eocene (22 localities), and (4) early Oligocene (15 localities). These occurrences all originate from the Cenozoic and indicate the importance of the pull-of-the-recent [47] in evaluating lepidopteran diversity patterns.

A total of 985 lepidopteran fossils have been assigned to 23 extant superfamilies (Figure 2; Table 2), of which the 214 affiliated with the Tineoidea were most numerous, followed by Papilionoidea (142), Noctuoidea (110), and Nepticuloidea (103). Nevertheless, fossil preservational type varies significantly by superfamily; in most cases, one or sometimes two preservation types were dominant (Table 1). The seven superfamilies of Bombycoidea, Cossoidea, Hepialoidea, Noctuoidea, Pterophoroidea, Pyraloidea and Zygaenoidea provided preservational types that predominantly or exclusively occurred in lacustrine deposits. By contrast, the nine superfamilies of Adeloidea, Gelechioidea, Lophocoronoidea, Micropterigoidea, Mnesarchaeoidea, Tineoidea, Thyridoidea, Tortricoidea and Yponomeutoidea were represented entirely or predominantly in amber and copal resins that typically originate from forested ecosystems. The three superfamilies of Gracillarioidea, Nepticuloidea and Tischerioidea were dominantly represented by leaf mines.

### Diversity trends

The family-level diversity of Lepidoptera increases significantly toward the recent [47], and the highest diversity values of the Pliocene–Pleistocene remain significantly lower than their extant family-level diversity (Figure 4). Our data show a relatively low linear correlation (Table 2, R^2^ = 0.729) between the increase in family diversity of Lepidoptera and geologic time, attributable to considerable Cenozoic diversity fluctuation for lepidopteran families. This relationship has a better fit under an exponential model (Table 2, R^2^ = 0.9027). The Trichoptera alone (Figure 4) and the Amphiesmenoptera of the Trichoptera + Lepidoptera (Figure 3) also exhibit a family-level diversity increase that is poorly fitted to a linear regression (Table 3, R^2^ = 0.8302 and 0.7138 respectively). By contrast, for the Hymenoptera and Diptera, family-level increases assume a linear trajectory (Figure 4 and Table 3, R^2^ = 0.9588 and 0.9109 respectively). The Coleoptera demonstrates that both linear and exponential models explain well their family-level diversity increase (Figure 4 and Table 3).

**Table 3:** **Linear and exponential regression equations (y) coefficients (R** ^**2**^ **) for Figures** **2** **and** **3**

	**Linear**		**Exponential**	
	**y**	**R** ^**2**^	**Y**	**R** ^**2**^
Lepidoptera (Labandeira, 1994)	2.3187x - 67.702	0.7343	0.0788e^0.1249x^	0.9292
Lepidoptera (Sohn et al., 2012)	2.0147x - 54.575	0.729	0.1886e^0.1083x^	0.9027
Lepidoptera + Trichoptera	1.695x - 23.963	0.7138	0.7045e^0.0903x^	0.9696
Trichoptera	0.4922x - 4.8454	0.8302	0.9301e^0.0627x^	0.9614
Coleoptera	2.4267x - 17.481	0.9417	6.2521e^0.0589x^	0.924
Diptera	2.1594x - 23.443	0.9109	1.9969e^0.0794x^	0.7833
Hymenoptera	2.0268x - 25.753	0.9588	1.6138e^0.081x^	0.7031

## Discussion

### Lepidopteran fossil abundance

It is generally considered that Lepidoptera are relatively scarce among insect fossils [1,11,21], and represent a Lagerstätten driven record consisting of deposits that are exceptionally well preserved or bear extremely abundant specimens [48]. This widely-accepted perception, however, is seldom based on actual counts of existing lepidopteran fossils. Kristensen and Skalski [10] were the first to provide figures of the total number of known lepidopteran fossils, which they estimated at 600 to 700 specimens. We calculated the number of existing lepidopteran fossils from the latest catalog [32] and arrived at 4,593 specimens. This number is somewhat more than seven times larger than of Kristensen and Skalski’s [10] estimate. Part of this significant increase is attributable to greater activity in finding new lepidopteran fossils since Kristensen and Skalski’s findings. For example, Rust [49,50] reported over 1,000 new lepidopteran fossils from the late Paleocene Fur Formation of Denmark that were not included in Kristensen and Skalski’s [10] account. Another possible cause for the increase is that Sohn et al. [32] included several historical collections which currently cannot be located and were not counted by Kristensen and Skalski [10]. Given these and other considerations, Kristensen and Skalski seem to have significantly underestimated the total number of the lepidopteran fossils.

### Lepidopteran taphonomy

In spite of the recent remarkable increase in the total number of lepidopteran fossils, the Lepidoptera appears considerably less abundant than the other, hyperdiverse insect orders, in particular the Coleoptera, Diptera and Hymenoptera. For example, lepidopteran inclusions in most amber deposits constitute less than 1% of whole-insect specimens [51]. This depauperate lepidopteran record apparently is due to their fragile bodies and wings [9]. In fact, actualistic taphonomic simulations of extant Lepidoptera suggest that their submerged bodies and wings are easily dismembered and undergo quick decomposition [52]. The buoyancy of their bodies due to water-resistant wing scales [53] results in exposure and encourages predation, thus rendering very unlikely the chances for fossilization in lacustrine deposits [21,54]. The proportional representation of Lepidoptera in amber appears low, as many Lepidoptera are strong fliers and apparently avoid being trapped in plant resin [19]. Most lepidopteran fossils are fragmentary, resulting in the absence of diagnostic characters useful for defining their taxonomic identity with certainty. This difficulty has led to the paucity of described lepidopteran fossils with convincing taxonomic evidence. Kristensen and Skalski [10] predicted that about one-third, or about 220 taxa, of all known lepidopteran fossils have been described and named. This estimated proportion of name-bearing lepidopteran fossils however is much less than one-third, and is attributable to new discoveries of fossils, which overwhelmingly were unidentifiable to any useful taxonomic level. Our data show that about 7% of the total fossil lepidopteran specimens (n = 236) have been formally described and named. This number of name-bearing fossil species is far less than, for example, Diptera which comprises 3,245 described fossil species [55].

Earlier examinations indicated that lepidopteran fossils occur principally as amber inclusions and larval leaf-mine compressions and impressions [21]. Our data suggest that compression–impression fossils and amber–copal inclusions collectively account for 92% of all lepidopteran specimens. Kristensen and Skalski [10] estimated that approximately 500 out of 650 fossils are preserved as amber or copal. This proportion is significantly different from our estimate that demonstrates compression–impression fossils are 12% more abundant than resin-originating body fossils (Figure 1A). In addition, the proportion of compression–impression fossils increases significantly when all trace fossils are included, especially as leaf mines are considerably more documented in fine-grained sediments than they are as rare inclusions in amber or copal (Figure 1B and 1C). This difference in representation appears partly due to more recent collecting activity, such as compression–impression material retrieved from the Danish Fur Formation. The third most frequent preservational type of lepidopteran fossils are sieved residues, corresponding to 7% of total specimens. Sieved residues are disarticulated cuticular sclerites or body fragments that originate from unconsolidated matrix, typically from late Pliocene to early Holocene deposits and are associated with relatively recent glacial–interglacial environments [56].

Trace fossils likely associated with Lepidoptera consist predominantly of leaf mines and larval cases (Figure 1B). These two types of trace fossils differ remarkably in preservational type and their occurrence in the sedimentary record. Leaf-mine fossils predominate as compressions or impressions of foliage, and almost never are present as leaf fossils in amber [57], a pattern reflecting the considerably greater foliar surface areas available in fine-grained slabs of sedimentary matrix, when compared to an amber record of miniscule, entombed leaf fragments. Alternatively, some mid Cenozoic deposits, such as Baltic Amber, contain a surprising abundance of larval cases, particularly psychid moths [58]. Other rare types of lepidopteran feeding damage include wood borings and external foliage feeding [59], although attribution to a lepidopteran culprit is rarely possible. Occasionally, fossilized larval frass, preserved as small coprolites, have been misidentified as seeds or even small fruits [60], although the surface features of such structures can readily be distinguished, separating the two [61]. These considerations suggest that a thorough review of seeds and other plant reproductive structures may reveal additional misidentifications, potentially increasing the proportion of taxonomically affiliated larval frass in the lepidopteran fossil record.

Lepidopteran fossils show extreme age bias toward the earlier Paleogene Period, accounting for about 80% of their total fossil occurrences (Figure 1C). A large proportion of compression–impression occurrences from only a few deposits are preserved during the Paleocene Epoch, especially late Paleocene (58.7–55.8 Ma: Table 1). The Eocene Epoch, especially the middle and late Eocene (48.6–33.9 Ma: Table 1), by contrast, has a high proportion of occurrences that represent varied preservational types originating from eleven, geographically disparate, major fossil localities that includes compression–impression material and especially amber. A small peak of occurrences during the Miocene Epoch notably corresponds to the highest number of fossil localities. Fossils from this interval represent a variety of preservational types, but are dominated by compression–impression fossils. The Quaternary Period also shows a small peak, predominantly comprising sieved residues. Generally, the numbers of fossil specimens and fossil localities are not congruent, except for the Eocene, the Miocene and the Pleistocene + Holocene intervals, separated by intervals representing a scarcity of lepidopteran fossils.

### Taxonomic composition of lepidopteran fossils

Labandeira [44] estimated that 63.4% of all extant insect families are represented by at least one occurrence in the fossil record. However, the compendium on which this estimate was made currently is outdated [45], and would require updates to provide a modern assessment. This percentage is high for major holometabolous orders, but for the Lepidoptera, the fossil capture rate of extant families was significantly lower, at 42.0%. This low percentage, derived from Labandeira’s data [44], shows that only 985, or 21.4% of total lepidopteran fossil specimens, have been placed into 23 of the 40 extant lepidopteran superfamilies (Table 2; Figure 1), for a capture rate of 57.5%. These taxonomically assigned fossils predominantly were amber–copal inclusions (38.4%), followed by compression–impression body fossils (19.0%), and leaf mines (16.6%). These proportions contrast significantly to the preservational composition of all lepidopteran fossils, reflecting that amber fossils are more amenable to superfamily-level identification than other preservational types. It is highly likely that the low capture rates of lepidopteran superfamilies (and families) resulted from interplay of the difficulty of identifying fossils, especially specimens from compression-impression material, and their poor fossil availability.

The representation of lepidopteran superfamilies in the fossil record varies considerably, and likely depends on biological peculiarities such as the habitat frequented, extent of geographically delimited population size, flight ability, and other mostly dispersal-related attributes of particular lineages. For example, relatively abundant fossils of Tineoidea often occur as inclusions in amber, with arboreal detritivorous and exophytic feeding patterns that provide opportunities for entrapment in plant resins. Leaf-mine fossils of Nepticuloidea also are strongly associated with an arboreal existence, but unlike tineoid taxa, feature herbivorous and endophytic feeding habits. Consequently, there is preferential occurrence of nepticuloids in compression–impression deposits. The fossil record of leaf-mining superfamilies are heavily dependent on expanses of foliar surfaces in stratal bedding planes, although identifications of leaf mine taxa have been questioned by some [10,21,62]. In taphonomically different settings, noctuoid fossils may have inflated abundances, since their preservation as scales, sclerites and other cuticular fragments in vertebrate gut contents and coprolites [63] can be taxonomically associated with the same individual prey item. The relatively large proportion of Papilionoidea fossils is surprising, given that this group accounts for only about 15% of the extant macrolepidopteran fauna [21]. This disproportionate fossil abundance likely is due to elevated anthropogenic interest, as is the case for extant butterflies, which encourages more attention toward identification and description of the fossil species of butterflies than of other lepidopteran groups. Fossils of the Bombycoidea, Cossoidea, Hepialoidea, Noctuoidea, Pterophoroidea, Pyraloidea and Zygaenoidea predominantly or exclusively are from sedimentary compressions. Members of these superfamilies, except for the Pterophoridae, possess relatively large body sizes and consequently have robust flight musculature, allowing for resistance to resin entrapment and thus explaining their rarity in amber. Among macrolepidopteran superfamilies, the Geometroidea are exceptional in having near equivalent numbers of specimens from fine-grained sedimentary matrices as well as fossil resins, although only a limited number of fossils are known for the group. Notably, microlepidopteran lineages are considerably enriched in amber deposits. The Gelechioidea, Tineoidea and Tortricoidea are relatively more abundant in amber than in fine-grained sedimentary matrices. These patterns of representation are consistent with Skalski’s [19] observation that two families, Tineidae and Oecophoridae (auct.), constitute approximately 30% of all lepidopteran inclusions in amber.

Our data show that the taxonomic representation in the lepidopteran fossil record is biased toward a few superfamilies, and is roughly proportional to their extant diversity, except for the better represented Papilionoidea. The fossils of each superfamily also are subject to preservational bias and, consequently, a distributional bias based on fossil age. These biases indicate that lepidopteran fossil data are very incomplete, and appropriate interpretation would require correction factors. For example, amber deposits predating the Late Cretaceous are very rare, limiting coverage of older lepidopteran history [64]. Such a geochronological limitation needs to be taken into account for interpreting the fossil record, especially of microlepidopteran superfamilies whose taxa are heavily entombed in amber.

### Lepidopteran diversity in the fossil record

Labandeira and Sepkoski [9] found that lepidopteran family diversity, when projected over geologic time, deviates from the expected pattern of a gradual and proportional increase toward their current diversities, typically displayed by other insect orders. As determined by Labandeira [44] and Ross et al. [65], the diversity increase of lepidopteran families is indeed nonlinear, significantly differing from other major holometabolous insect orders which exhibit gradual, linear increases through time (Figure 4). This deviation seems to be related to the low fossil capture rate of lepidopteran families. We tested if the recent increase in the number of identified lepidopteran fossils [31] would negate such a deviation (Table 3). Our linear regression result yielded a slightly lower value (R^2^ = 0.729) than one estimated for Labandeira [44]. Therefore, despite recent updates to the lepidopteran fossil record, their unusual evolutionary pattern of family-level diversity evolution still holds. This absence of significant change indicates that most additional fossils since Labandeira [44] were ones where family-level assignments already had a fossil record or otherwise lacked a family assignment. Indeed, the differences between Labandeira’s [44] and our estimate are principally attributable to changes in the family-level classification system of the Lepidoptera. The unusual family-level diversity increase in the Lepidoptera seems to be a more general feature of the Amphiesmenoptera, as our data incorporating the Trichoptera into the Lepidoptera resulted in a further lowering of the linear regression estimate (Table 3).

The fluctuation in lepidopteran family-level diversity is better described by exponential models (Table 3), rather than by linear regression. Either solution supports a putative recent diversification of the Lepidoptera [8,21,28,29]. However, this pattern requires careful interpretation. For example, in comparison to other insect orders, the Lepidoptera exhibits weak family-level diversity peaks during the Paleocene (ca 65.5–55.8 Ma) and Miocene (ca 23.0–5.3 Ma). It is known that many lepidopteran fossils are recorded from these strata, such as the late Paleocene Fur Formation compressions and early Miocene Dominican amber. However, these elevated diversities are better explained by the pull-of-the-recent [47], which is a phenomenon whereby a more complete fossil record toward the present day also predilects for a greater taxonomic representation of fossil taxa. Rainford et al. [46] observed more recent shifts in the diversification of Lepidoptera, corresponding to the emergence of major clades such as Glossata, Ditrysia and the redefined Obtectomera. This may support the ‘key innovation’ hypothesis, which highlights the emergence of evolutionary novelties that drive taxonomic richness [66]. Such a pattern, however, was not observed from the diversity increase of lepidopteran families plotted from our study (Figure 2) and also that of Ross et al. [65], which traced the lepidopteran family diversity using origination and extinction rates. A complicating factor is the need for a greater awareness of the existence of lepidopteran specimens that remain unidentified at least to the family-level. Future studies attempting to more accurately resolve the taxonomic identities of unstudied lepidopteran fossils likely will fill existing gaps in the fossil record. Also, it is likely that the lepidopteran fossil record will increasingly track a more familiar linear increase in family-level diversity, as demonstrated for other insect orders.

### Lepidopteran divergence in the fossil record

Figure 2 depicts fossil occurrences of lepidopteran superfamily-level lineages from a current working hypothesis of lepidopteran phylogeny [40]. Similar, but morphology-based phylogenies, calibrated by key fossil occurrences, were constructed by Kozlov [67], Labandeira et al. [68], Grimaldi [62], and Grimaldi and Engel [21]. Lately, molecular phylogeny-based divergence time estimates became available for the entirety of the Lepidoptera [15]. Most of those studies dated the origin of Lepidoptera approximately to the Sinemurian Stage (196.5–189.6 Ma) of the Early Jurassic (Figure 2: occurrence 1), based on the fossil, *Archaeolepis mane* (Whalley, 1985), the earliest known lepidopteran. Wahlberg et al. [15], however, pushed the age of the crown clade of Lepidoptera back to as old as 215 Ma, the mid Late Triassic. Thereafter, during the later Early Jurassic to earlier Middle Jurassic, several lineages with robust mandibulate mouthparts originated in succession [22-24,67,69], eventually giving rise to the Glossata which are characterized by fluid-imbibing, siphonate mouthparts [13].

Labandeira et al. [68] dated the divergence of Glossata and earlier clades at ca. 160 Ma, based on putative early lepidopteran specimens, including 180 million-year-old mandibulate forms from Grimmen [22], and ca. 155 million-year-old specimens from Karatau (Kazakhstan), particularly the basal moth *Protolepis cuprealata* Kozlov 1989, that controversially may have possessed a short siphon for imbibition of fluid food [10] (contra [21]). This hypothesis considers an initial short fuse followed by diversification of basal lepidopteran groups during the first 25 million years of the lepidopteran fossil record. In contrast, Grimaldi and Engel [21] propose an alternative hypothesis, indicating that such divergence events occurred considerably later, centered in the mid Late Jurassic to Berriasian, the earliest stage of the Cretaceous, and perhaps coincident with initial angiosperm diversification [62,70]. Grimaldi and Engel [21] considered an undescribed larva in Lebanese amber (about 130 Ma, mid Early Cretaceous) as the earliest Glossata. This view would maintain an initial 35 million-year interval of stasis, or a long fuse, followed by a relatively sudden, 15 million-year interval of rapid cladogenesis from 155 to 140 Ma, toward the end of which the Glossata evolved. Imada et al. [71] estimated the divergence of Zeugloptera from Glossata with molecular data which spanned a 170 to 135 million-year interval. Wahlberg et al. [15] estimated Glossata evolved from non-glossatan lineages as early as 212 Ma, taking this lineage to the mid Late Triassic. Currently, there exists no fossil evidence verifying such early Triassic divergence of Glossata from more plesiotypic, mandibulate lineages.

The Late Jurassic origin of Glossata as asserted by Grimaldi and Engel [21] necessitates the explosive diversification of the ditrysian lineages during a time interval spanning the Late Cretaceous and Paleocene (100–55 Ma), which is significantly later than the initial angiosperm radiation [72,73]. This may imply that the Lepidoptera colonized, rather than coevolved with, already diversified angiosperms. By contrast, Labandeira et al. [68] suggested that the divergence of Ditrysia may predate the angiosperm radiation. A divergence-time estimate of Ditrysia (156.7 Ma) by Wahlberg et al. [15] may support the hypothesis of Labandeira et al. [68].

Grimaldi and Engel [21] assumed the more recent occurrence of major lepidopteran clades, when compared to the estimates from Wahlberg et al. [15], based on a relaxed molecular-clock method. These differences may be due to two recent advancements in the evolutionary studies of Lepidoptera. First, Grimaldi and Engel [21] adjusted the fossil occurrence of lepidopteran superfamilies according to the phylogeny constructed by Kristensen and Skalski [10]. Recent molecular studies [38-41] critically revised Kristensen and Skalski’s phylogeny with changes in the systematic positions of some superfamilies, for example, Gelechioidea and Papilionoidea. Revised placements of these two superfamilies may lead to differences between the accounts of Grimaldi and Engel [21] and Wahlberg et al. [15] in the divergence-time estimates of the Apoditrysia and Macroheterocera respectively. Second, Sohn et al. [32] published a comprehensive fossil catalog of Lepidoptera which was not available to Grimaldi and Engel [21]. Primarily using the catalog, Wahlberg et al. [15] included three fossil calibration points that Grimaldi and Engel [21] did not consider. These fossil occurrences are either newly discovered, postdating Grimaldi and Engel [21], or alternatively were calibrations that lacked diagnostic characters not considered by Grimaldi and Engel [21].

Kozlov et al. [11] observed that the relative dominance of microlepidopteran over macrolepidopteran fossils in the Eocene was reversed during the late Oligocene and early Miocene. This observation raises the possibility that macrolepidopteran diversity increased only after the Eocene. We did not recover such a pattern of replacement in our data, which includes more lepidopteran fossil specimens than those used by Kozlov et al. [11].

### Implications of lepidopteran fossil record to their divergence-time estimates

The Lepidoptera have been considered conventionally as consisting of a depauperate fossil record. Although such a perception often was based on the sparseness of lepidopteran fossils, there have been no studies that systematically evaluated the record with specimen abundance data based on locality, geologic age, higher-level taxa, preservational mode, and other relevant variables. We scrutinized the entire lepidopteran fossil record with a systematic approach in mind and found three major taphonomic or research biases.

The first perception is that the most common preservational mode characterizing lepidopteran identifications is amber. This type of preservation bias could be problematic in that amber fossils cover a shorter time window than compression-impression fossils. The oldest insect-bearing ambers extend only to about 130 Ma [74], effectively rendering older occurrences of fossils available only as compressions or impressions [35,51] (but see [75]). Consequently, family-level diversity of Lepidoptera prior to the Late Cretaceous is likely to be underestimated because of the absence of available amber fossil deposits with insect inclusions.

A second factor is that lepidopteran fossil occurrences in general are extremely biased toward the Paleogene Period. This enrichment may be due to the increased, idiosyncratic, preservational potential of lepidopteran fossils during the Paleogene, or possibly related to the pull-of-the-recent [76]. Such a bias would draw downward the occurrence of superfamilies on both sides of the Paleogene, causing the appearance of explosive diversification event rather than a more likely dramatic increase in preservational potential.

Last, the availability and density of fossil occurrences for establishing the presence of lepidopteran superfamilies appears highly variable across time, habitats and lineages. Such biases require that divergences of several superfamily-level lineages lack relevant fossils for establishment in the fossil record, rather than be inferred from sister groups with identifiable fossils or even cladogenetically related, more distant lineages. These three biases often are interrelated, and collectively present a sporadic and incomplete record, as shown by the prevalence of ghost lineages occupying dotted vertical lines that lack fossils in Figure 2.

Figure 2 also shows that the fossil records of several lepidopteran superfamilies deviate substantively from the molecular estimates of their divergences. Fossil occurrences are almost always later than their molecular divergence estimates [72,77], as it is extremely unlikely that fossils capture the moments of initial lineage divergence. The extent of such deviations, however varied, depends on the temporal density of fossil occurrences in lepidopteran superfamilies. For example, a few superfamilies such as Hepialoidea, Tischerioidea, Sesioidea, and Bombycoidea have their earliest fossil occurrences mismatched with their molecular-estimated divergences by more than 35 million years. The fossils of these superfamiles are either sparse or subject to uncertainty in identification. Among the relatively fossil-dense superfamilies, the earliest fossil occurrence and the molecular divergence estimate are mismatched for Papilionoidea and Pyraloidea, but both are closely matched in Adeloidea, Gracillarioidea, Gelechioidea, Geometroidea and Noctuoidea. Two superfamilies, Eriocranioidea and Nepticuloidea, displayed earlier fossil occurrences than that suggested by Wahlberg et al. [15]. Interestingly, these body-fossil occurrences are predated by putative leaf mine traces—a common feature of the fossil record [34]. Reliable divergence-time estimation of molecular phylogenies depends on the quality of the fossil record, and a poor fossil record of Lepidoptera may be of minimal use for such analyses. Recent progress in molecular dating methods nevertheless would allow for establishment of uncertainties in fossil calibrations [18,76]. Our study may address the effects of sampling the fossil record, and assist in resolving discrepancies between molecularly-based estimates and paleontological evidence [78].

## Conclusions

The fossil record of Lepidoptera has long been considered significantly incomplete, limiting its relevance to their evolutionary biology [1]. Nonetheless, divergence time estimation depends heavily on fossil calibrations that have become routine in recent molecular-phylogenetic studies of Lepidoptera. Our overview has characterized the lepidopteran fossil record at the ordinal- and superfamilial levels through examination of total abundance, preservational type composition, age distribution and other factors. From these characterizations, we identified three taphonomic or research biases involved in the existing data as the following:

i.) A large proportion of the reliably identified lepidopteran fossils are preserved in amber and copal covering a shorter geologic time window than compression or impression fossils, the latter of which constitute the most abundant preservation type for lepidopteran fossils.
ii.) Lepidopteran fossil record shows a strong age bias toward the earlier Paleogene, which indicates a strong effect of the pull-of-the-recent.
iii.) Lepidopteran taxa vary in fossil availability based on their membership in particular superfamilies, a bias that depends significantly on their taphonomic context.

Our overview also reveals that about 78% of lepidopteran fossils remain unidentified and most fossils representing the earliest occurrence of a given lineage are subject to issues regarding identification. Plots of lepidopteran diversity and phylogeny through time suggest that a high proportion of their evolutionary history remains undetected in the fossil record. Therefore, we recommend that future molecular dating studies of Lepidoptera incorporate these possible sources of error from fossil specimens into their analyses.

## Additional file

Additional file 1:
**The list of fossils cited in Figure** **2** **with the literature sources of the fossil occurrences.**

## Abbreviation

Ma: Millions of years ago
