# Supplementary material for: The fossil record and taphonomy of butterflies and moths (Insecta, Lepidoptera): implications for evolutionary diversity and divergence-time estimates
Source: BMC Evol Biol. 2015 Feb 4;15(1):12. doi: 10.1186/s12862-015-0290-8 (PMC4326409; doi:10.1186/s12862-015-0290-8)
Supplement: Additional file 1: — The list of fossils cited in Figure 2 with the literature sources of the fossil occurrences. [file 12862_2015_290_MOESM1_ESM.pdf]

## Appendix

The following is a list of fossil occurrences and associated literature sources for Figure 4. For brevity, only taxon names and locality information are shown. This is not an exhaustive list. Due to graphical limitations, not all relevant deposits were included below or in Figure 4. More expansive accounts and literature sources for taxa in each record is provided by Sohn et al. (2012).

1. The archaeolepid, *Archaeolepis mane*, from a calcareous flatstone of Dorset, United Kingdom (Whalley, 1985), the earliest, reliably-identified lepidopteran.

2. Two undescribed Lepidoptera, related to the Micropterigidae, from the Grüne Series of Grimmen, Germany (Ansorge, 2002).

3. *Auliepterix mirabilis*, a micropterigoid specimen, from the Karabastau Formation, Kazakhstan (Kozlov, 1989).

4. *Auliepterix minima*, a micropterigoid specimen, from the Archangel Formation of Mongolia (Kozlov, 1989).

5. The micropterigid, *Palaeosabatinca zherichini*, from the Zaza Formation of Asian Russia (Kozlov, 1988).

6. *Parasabatinca aftimacrai* from the Grès de Base Formation, Lebanon (Whalley, 1978); *P. caldasae* from the Crato Formation, Brazil (Martins-Neto & Vulcano, 1989); *Sabatinca perveta* (Cockerell, 1919) from Burmese amber of an unnamed formation (Rasnitsyn and Ross, 2000), in northern Myanmar; and an indeterminate genus in Álava Amber from the Nograro Formation, Spain (Martínez-Delclós et al., 1999).

7. Micropterigid wing scale in inclusions from Aquitanian Amber of France (Kühne et al., 1973).

6. The micropterigid *Moleropterix kalbei*, from the Fur Formation of Denmark (Engel & Kinzelbach, 2008).

9. Four described micropterigid species, *Baltimartyria proavittella*, *B. rasnitsyni*, *Micropterix gertraudae* and *M. immensipalpa*, in Baltic Amber from the Prussian Formation of the Baltic Region (Rebel, 1936; Kusnezov, 1941; Kurz & Kurz, 2010; Mey, 2011).

10. The micropterigid, *Micropterix angelica*, from the Bouldnor Formation of the United Kingdom (Jarzembowski, 1980).

11. The glossatan, *Protolepis cuprealata*, from the Karabastau Formation of Kazakhstan (Kozlov, 1989).

12. An undescribed eriocranoid specimen from the Karabastau Formation of Kazakhstan (Kozlov et al., 2002).

13. An undescribed eriocranoid specimen in Burmese Amber from an unnamed formation, northern Myanmar (Skalski, 1990b).

14. An undescribed specimen in Baltic Amber from the Prussian Formation, Baltic Region (Skalski, 1990b).

15. The eriocranoid, *Eriocranites hercynicus*, from lake sediments of Willershausen in Germany (Kernbach, 1967); and undescribed leaf mines from the Payette Formation, United States (Opler, 1973).

16. An undescribed lophocoronid specimen in Taimyr Amber from the Kheta Formation, European Russia (Skalski, 1979a).

17. An undescribed mnesarchaeoid or hepialoid specimen in Taimyr Amber of the Kheta Formation, from European Russia (Zherikhin & Sukacheva, 1973).

18.The hepialioid, *Prohepialus incertus*, from the "cinerites" of Puy-de-Dôme, France (Piton, 1940).

19. Undescribed mnesarchaoid or hepialoid specimens from the Bouldnor Formation (Jarzembowski, 1976), and the Glen Afton mine (Evans, 1931), of the United Kingdom.

20. The hepialoid specimens *Protohepialus incertus*, from the Puente Formation of California, USA (Skalski, 1990a); and *Oiophassus nycterus*, from the Shanwang Formation, of Shandong, China (Zhang, 1989).

21. A possible undescribed nepticulid leaf mine, from the Karabastau Formation, Kazakhstan (Skalski, 1979a).

22. Several undescribed leaf mines, attributed to the Nepticulidae, from the Battle Camp Formation of Queensland, Australia (Rozefelds, 1988).

23. Undescribed nepticulid leaf mines from the Dakota Formation, Kansas and Nebraska, USA (Stephenson, 1991; Labandeira et al., 1994); a *Stigmellites araliae* leaf mine from the Perucher Formation of the Czech Republic (Fritsch, 1982).

24. Five described morphospecies (*Stigmellites kzyldzharicus*, *S. samsonovi*, *S. serpentina*, *S. sharovi*, *S. tyshchenkoi*) from the Beleuty Formation of the Kyzl-Ordinsky Region, Kazakhstan (Kozlov, 1988).

25. Undescribed nepticulid leaf mines from the Meeteetsee Formation of Wyoming, USA (Labandeira, 2002b), and undescribed leaf mines from the Hell Creek Formation of North Dakota, USA (Labandeira et al., 2002b).

26. The nepticulid leaf mines *Stigmellites centennis* and *S. gossi* from the Reading Formation (Jarzembowski, 1989), of late Paleocene age from the United Kingdom; and *Stigmellites messelensis* from the Messel Formation (Straus, 1976), of middle Eocene age, from Germany.

27. *Stigmellites balticus* from Baltic amber of the Prussian Formation (Kozlov, 1988); undescribed leaf mines from the Chuckanut and Klondike Mountain Formations (Labandeira, 2002a) of Washington State, USA; and nepticulid leaf mines from the Branksome Sand Formation, United Kingdom (Jarzembowski, 1995).

28. Undescribed nepticulid specimens from the Bouldnor Formation, United Kingdom (Jarzembowski, 1980).

29. The nepticulid leaf mine *Stigmellites fossilis* from the Rott Formation, Germany (Heyden, 1862; Kozlov, 1988); and *Stigmella* (?) *almeidae* from the Tremembé Formation, Brazil (Martins-Neto, 1989).

30. Undescribed nepticulid specimens from bituminous rhythmites at the “La Rinconada” site, Spain (Peñalver and Delclòs, 2004); undescribed nepticulid mines (Liebhold et al., 1982) from the Trapper Creek Formation, Idaho, USA; and various other North American middle Miocene fossil sites (Opler, 1973) of the Pacific Interior, USA.

31. Five described nepticulid species from several fossil sites: *Stigmella ulmivora*, *Stigmellites carpiniorentalis*, *Stigmellites heringi*, *Stigmellites pliotityrellus* and *Stigmellites zelkoviae,* all of the late Pliocene lake sediments of Willershausen, Germany (Kernbach, 1967; Straus, 1977).

32. Adeloid wing scales from Lebanese Amber of the Grès de Basa Formation, Lebanon (Whalley, 1978).

33. Feeding trace fossils from the Upper Hatira Formation, Israel (Krassilov and Shuklina, 2008); an undescribed incurvariid specimen from Taimyr Amber of the Kheta Formation, European Russia (Skalski, 1979a).

34. Stereotypical incurvariid leaf-case fossils of earlier middle Eocene age from the Green River Formation of Colorado (Labandeira, 1998), Klondike Mountain Formation of Washington (Labandeira, 2002a), and the Messel Formation of Germany (Labandeira et al., 2007).

35. Eight described adeloid species of later middle Eocene age in Baltic Amber from the Prussian Formation (Rebel, 1934; Rebel, 1936; Kozlov, 1987): *Adela kuznetzovi* and *A. similis*; *Adelites acutitarsellus*, *A. purpurascens*, *A. serraticornellus*, *A. electreellus*; *Prophalonia gigas*; and *Incurvarites alienellus*.

36. Incurvariid leaf mines from the Willershausen lake deposits of Germany (Straus, 1977).

37. An undescribed tischeriid leaf mine from the Ripley Formation of Tennessee, USA (Stephenson, 1991).

38. An undescribed larval case specimen of *Eudarcia* from Baltic Amber of the Prussian Formation, in Germany, Poland, Russia and adjacent countries (Sobczyk and Kobbert, 2009).

39. Three described and several undescribed psychid specimens (Menge, 1856; Rebel, 1934; Kozlov, 1988; Sobczyk and Kobbert, 2009) from Baltic Amber of the Prussian Formation, in Germany, Poland, Russia and adjacent countries.

40. *Adelopsyche frustrans* from the Florissant Formation of Colorado, USA (Cockerell, 1926).

41. Feeding mark fossil supposedly made by a psychid (Lewis, 1976) of later Oligocene from the Renova Formation of Montana, USA.

42. The tineoid larval case *Psychites pineellus* from the "Molasseformatien" of Oeningen, Germany (Heer, 1849).

43. Undescribed specimens from New Jersey Amber of the Raritan Formation, USA (Grimaldi and Nascimbene, 2010).

44. Approximately twenty described and several undescribed tineoid specimens (Menge, 1856; Rebel, 1934; Kusnezov, 1941; Skalski, 1974, 1977; Jarzembowski, 1980; Kozlov, 1987, 1988) from Baltic Amber of the Prussian Formation, in Germany, Poland, Russia and adjacent countries.

45. The tineoids *Paratriaxomasia solentensis* from the Bouldnor Formation, United Kingdom (Jarzembowski, 1980).

46. Undescribed tineoid trace- and body fossils (Hurd et al., 1962; Poinar, 1992) of later Oligocene to earlier Miocene age from the Simojovel Formation of Mexico.

47. Undescribed body fossils (Kristensen and Skalski, 1998; Grimaldi and Engel, 2005) in Dominican Amber, from the La Toca Formation, Dominican Republic.

48. Undescribed gracillariid specimen from Burmese Amber in an unnamed formation, northern Myanmar (Ross et al., 2010).

49. Phyllocnistine and gracillarine leaf-mine fossils (Chambers, 1882; Stephenson, 1991; Labandeira et al., 1994), from the Dakota Formation, Kansas and Nebraska, USA.

50. The gracillarioid *Bucculatrix platani* from the Beleuty Formation of Kazakhstan (Kozlov, 1988); undescribed leaf mines from the Frontier Formation of Wyoming, USA (Knowlton, 1917); and leaf mines from the Ora Formation, Israel (Krassilov and Shuklina, 2008).

51. Undescribed gracillarioid leaf mines from the Hell Creek Formation of North Dakota, USA (Labandeira et al., 2002a, 2002b).

52. An undescribed bucculatricid leaf mine from the Wind River Formation, Wyoming, USA (Hickey and Hodges, 1975); and gracillariid leaf mines from the Tufulitas Laguna del Hunco Formation, southern Argentina (Wilf et al., 2005).

53. The body fossil *Gracillariites lithuanicus* and *G. mixtus* from Baltic Amber of the Prussian Formation, Baltic Region (Kozlov, 1987); and undescribed leaf mines (Stephenson, 1991; Jarzembowski, 1995; Labandeira, 2002a), from several middle Eocene deposits of the United Kingdom and USA.

54. Undescribed leaf mines from the Florissant Formation of Colorado, USA (Opler, 1982), and the Bouldnor Formation of the United Kingdom (Jarzembowski, 1980).

55. The leaf mine *Phyllonorycter* (?) *oliveirae* from the Tremembé Formation of southern Brazil (Martins-Neto, 1989); and a leaf mine from Mexican Amber of the Simojovel Formation, Mexico (Poinar and Brown, 2002).

56. Undescribed gracillarioid leaf mines of *Acrocercops*, *Bucculatrix*, *Cameraria* and *Lithocolletis* from several Miocene deposits (Buffalo Canyon, Esmeralda, Latah, Payette, Savage Canyon and Trout Creek Formations) of Idaho, Nevada, Oregon and Washington, western USA (Opler, 1973; Lewis, 1985).

57. Two gracillaroid leaf mines attributed to the extant taxa, *Phyllonorycter maestingella* and *Bucculatrix thoracella*, from the Willershausen lake beds of Germany (Straus, 1977).

58. A putative yponomeutoid leaf mine assigned to the Praydidae from the Dakota Formation, Kansas and Nebraska, USA (Stephenson, 1991).

59. Nine described yponomeutoid species: *Argyresthites balticellus*, *A. succinellus*, *Plutellites acutipenellus*, *P. inversellus*, *P. minorellus*, *P. tenebricus*, *Epinomeuta truncatipennella*, *Scythropites balticellus* and *Prolyonetia cockerelli*, in Baltic Amber from the Prussian Formation of the Baltic Region (Rebel, 1934, 1936; Kusnezov, 1941; Skalski, 1976b, 1977; Kozlov, 1988).

60. Undescribed yponomeutoid specimens of late Eocene to early Oligocene age from the Bouldnor Formation (Jarzembowski, 1980) of the United Kingdom, and from Sicilian Amber (Skalski and Veggiani, 1990), Italy.

61. An undescribed yponomeutoid specimen from East African copal of Zanzibar, Tanzania (Skalski, 1976a).

62. Six described tortricid species: *Electresia zalesskii*; *Tortricibaltia diakonoffi*; *Tortricidrosis inclusa*; *Spatalistiforma submerge*; *Tortricites sadilenkoi*, *T. skalskii*, in Baltic Amber of the Prussian Formation, from the Baltic Region (Kusnezov, 1941; Skalski, 1973a, 1992; Kozlov, 1988).

63. The tortricid species *Tortricites destructus* and *T. florissantanus* from the Florissant Formation of Colorado, USA (Cockerell, 1916; Skalski, 1992; Meyer, 2003).

64. An undescribed tortricid specimen in Mexican Amber from the Simojovel Formation (Skalski, 1973a); and *Polyvena horatis* Poinar and Brown, 1993, and other undescribed fossils (Poinar, 1992; Grimaldi and Engel, 2005) from somewhat younger Dominican Amber, Dominican Republic.

65. Tortricid larval feeding damage attributed to the extant *Retinia resinella* from the Piionso Moor, Finland (Koponen and Nuorteva, 1973).

66. An undescribed sesiid specimen from the “laminites lacustres” of Aix-en-Provence, France (de Serres, 1829; Rasnitsyn and Zherikhin, 2002).

67. Undescribed zygaeniid material, assigned to the Procridinae from the Messel Formation, Germany (McNamara et al., 2011).

68. The zygaeniid *Neurosymploca* (?) *oligocenica* from the Calcaires de Montfuron or Calcaires de Vachères Formation of Céreste, France (Fernández-Rubio and Nel, 2000).

69. An undescribed zygaeniid specimen from the “laminites lacustres” of Aix-en-Provence, France (Leestmans, 1983); the zygaeniids *Zygaena* (?) *miocaenica* and *Zygaenites controversus* from the “dysodile beds” of Randecker Maar, Germany (Burgeff, 1951); and *Zygaena* (?) *turolensis* from the “bituminous rhythmites” of Rubielos de Mora, Spain

(Fernández-Rubio et al., 1991; Fernández-Rubio and Peñalver, 1994; Peñalver and Engel, 2006).

70. Undescribed limacodid specimen, cf. *Limacodes*, from the oil sands of Trinidad (Blair, 1927).

71. Cossoid cuticular fragments from the Messel Formation, Germany (Richter and Storch, 1980).

72. The cossid *Gurnetia durranti* from the Bouldnor Formation of the United Kingdom (Jarzembowski, 1980); and the castniid *Dominickus castnioides* from the Florissant Formation of Colorado, USA (Tindale, 1985).

73. Two cossid species, *Kleopathra*, *nemogypsia* and *K. noctodiva*, from the Tremembé Formation, Brazil (Martins-Neto, 1998a).

74. Undescribed gelechiid specimens in Burmese Amber from an unnamed formation in northern Myanmar (Ross et al., 2010).

75. Possible gelechiid leaf mines from the Dakota Formation, Kansas and Nebraska, USA (Stephenson, 1991).

76. Undescribed coleophorid leaf mines from the Upper Hatira Formation of Israel (Krassilov, 2007).

77. The elachistid *Hexerites primalis* from the Green River Formation of Colorado, USA (Kozlov, 1988).

78. Twenty five described gelechioid species, assigned to cf. *Anybia*, *Borkhausenites*, *Epiborkhausenites*, *Glesseumeyrickia*, cf. *Hofmannophila*, *Microperittia*, *Neoborkhausenites*, *Oecophorinites*, *Palaeodepressaria*, *Palaeoelachista*, *Paraborkhausenites*, *Praemendesia*, in Baltic amber from the Prussian Formation, Baltic Region (Rebel, 1934, 1936; Kusnezov, 1941; Skalski, 1973b, 1976b, 1977, 1979b; Kozlov, 1987; Kupryjanowicz, 2001).

79. The elachastid *Ethmia* (?) *mortuella* from the Florissant Formation of Colorado, USA (Kozlov, 1988; Meyer, 2003).

80. Several undescribed gelechioid specimens in Mexican amber from the Simojovel Formation (Hurd et al., 1962; Skalski, 1976b; Poinar, 1992); and several other gelechioid specimens in Dominican Amber from the La Toca Formation, Dominican Republic (Skalski, 1990b; Poinar, 1992; Grimaldi and Engel, 2005; Peñalver and Grimaldi, 2006).

81. Undescribed gelechiid and coleophorid leaf mines of late Miocene to late Pliocene age in Romania, Germany and Nevada, USA (Opler, 1973; Straus, 1977; Givulescu, 1984), some of which were attributed to the extant taxa, such as *Recurvaria nanella*.

82. An undescribed thyridid close to the extant *Rhodoneura* in Baltic Amber, from the Prussian Formation of the Baltic Region (Skalski, 1985).

83. An undescribed copromorphid species in Baltic Amber from the Prussian Formation of the Baltic Region (Skalski, 1990b).

84. The copromorphid, *Copromorpha fossilis* from the Bouldnor Formation, United Kingdom (Jarzembowski, 1980).

85. The pterophoroid body fossil, *Merrifieldia oligocenicus*, from the "laminites lacustres" of Aix-en-Provence, France (Bigot et al., 1986).

86. An undescribed pterophoroid body fossil from the "cinerites" deposit of Puy-de-Dôme, France (Piton, 1936).

87. The oldest described papilionoids, assigned to the Hesperiidae (Andersen & Andersen, 1996; Bonde et al., 2008), from the Fur and Ølst Formations of Denmark (Larsson, 1975).

88. Three described papilionoid species, *Praepapilio colorado*, *P. gracilis*; *Riodinella nympha* from the Green River Formation of Colorado, USA (Durden and Rose, 1978).

89. Undescribed papilionoid specimens in Baltic Amber from the Prussian Formation of the Baltic Region (Gravenhorst, 1835).

90. Thirteen described species, including the noted *Prodryas persephone* from the late Eocene Florissant Formation, Colorado, USA (Scudder, 1878); *Lithopsyche antiqua* and *Nymphalites zeuneri* from the Bouldnor Formation, United Kingdom (Jarzembowski, 1980); *Lethe* (?) *corbieri* and *Pseudoneorina coulleti* from the "laminites lacustres" of Céreste, France (Nel et al., 1993; Nel and Descimon, 1994); and *Thanatites vetulus* from the Rott Formation of Germany (Scudder, 1875).

91. Eight described papilionoid species, *Pamphilites abditus*, *Aquisextana irenaei*, *Lethites reynesii*, *Neorinopis sepulta*, *Thaites ruminianus*, and *Coliates proserpina*, from the "laminites lacustres" of Aix-en-Provence, France (Scudder, 1875; Théobald, 1937; Nel and Nel, 1986; Nel et al., 1993); and *Archaeolycorea ferreirai* and *Neorinella garciae* from the Trembembé Formation of São Paulo, Brazil (Martins-Neto, 1989; Martins-Neto et al., 1993).

92. Six described papilionoid species: *Voltinia dramba* and *Dynamine alexae* in Dominican Amber from the La Toca Formation, Dominican Republic (Hall et al., 2004; Peñalver and

Grimaldi, 2006); *Mylothrites pluto*, *Nymphalites atavus* and *Pontia freyeri* from a lignite deposit in Radoboj, Croatia (Heer, 1849; Scudder, 1875; Rasnitsyn and Zherikhin, 2002); *Miopieris talboti* from the "dysodile beds" of Randecker Maar, Germany (Zeuner, 1942); and *Aglais karaganica* from the Karagan Horizon of the Caucasus, Russia (Nekrutenko, 1965).

93. Late Pleistocene papilionoid specimens identified as extant butterfly species: *Andronymus neander*, *Charaxes candiope* and *Belenois crawshayi* from East African copal of Zanzibar, Tanzania (Skalski, 1976b).

94. An undescribed pyraloid specimen from the Fur Formation of Denmark (Bonde et al., 2008).

95. The pyraloid, *Glendotricha olgae* in Baltic Amber from the Prussian Formation of the Baltic Region (Kusnezov, 1941).

96. The pyraloid, *Pyralites preecei*, from the Bouldnor Formation, United Kingdom (Jarzembowski, 1980).

97. The pyraloids *Petisca dryellina* from the Tremembé Formation of São Paulo, Brazil (Martins-Neto, 1998b); and *Pyralites obscurus* from the "laminites lacustres" of Aix-en-Provence, France (Heer, 1856; Jarzembowski, 1980).

98. The pyraloid *Gallerites keleri* from lacustrine deposits at Willershausen, Germany (Kernbach, 1967).

99. An undescribed geometroid specimen from the Broken River Formation, North Island, New Zealand (Harris and Raine, 2002).

100. An undescribed geometroid specimen from the Klondike Mountain Formation of Washington, USA (Lewis, 1992).

101. Two geometroid specimens, *Geometridites larentiiformis*, from Bouldnor Formation, United Kingdom (Jarzembowski, 1980); and *Hydriomena* (?) *protrita* from the Florissant Formation of Colorado, USA (Cockerell, 1922).

102. An undescribed geometroid specimen from the "laminites lacustres" of Aix-en-Provence, France (Heer, 1961); and two species of *Phalaenites*, *obsoletus* and *crenatus* in a lignite deposit from Radoboj, Croatia (Heer, 1849; Rasnitsyn & Zherikhin, 2002).

103. The geometrid, *Problongos baudiliensis* from a diatomite of Saint-Bauzile, France (Mérit and Mérit, 2008).

104. A specimen of the extant *Hyperythra lutea* from East African copal of Zanzibar, Tanzania (Kozlov, 1988), and other geometroid material from the late Pliocene to late Pleistocene,

including *Geometrides jordani* and *G. repens* from the lacustrine deposits of Willershausen, Germany (Kernbach, 1967); and an indeterminate pupa of the Fossil Insect Research Group for Nojiri-ko Excavation (1990), from the Nojiri-ko Formation of Nagano Prefecture, Japan.

105. Doubtful silicified eggs, from the Magothy Formation of Massachusetts, USA (Gall and Tiffney, 1983; Kristensen and Skalski, 1998).

106. An undescribed noctuid specimen from the Fur Formation of Denmark (Bonde et al., 2008).

107. Undescribed noctuoid specimens from the Klondike Mountain Formation (Joseph, 1986), of Washington, USA; an indeterminate noctuoid from the Green River Formation of Wyoming, USA (Scudder, 1867).

108. Undescribed noctuoid specimens, including adults and larval frass in Baltic Amber from the Prussian Formation, Germany (Klebs, 1890, Bachofen-Echt, 1949; Nuorteva and Kinnunen, 2008); and a noctuid wing from the Allenby Formation of British Columbia, Canada (Douglas and Stockey, 1996).

109. The noctuioid *Noctuites incertissimus* from the “cinerites” of Puy-de-Dôme, France (Oustalet, 1870); the arctiid *Oligamatites martynovi* from Kazakhstan (Kusnezov, 1928); the catocalinid *Philodarchia cigana* from the Tremembé Formation of São Paulo, Brazil (Martins-Neto, 1998a); and the noctuoid *Noctuites deperditus* from the “laminites lacustres” of Aix-en-Provence, France (Heer, 1856; Kozlov, 1988).

110. There are approximately 12 early to middle Miocene specimens of noctuids: the seven described noctuoid species, *Noctuites caucasicus*, *N. kaspievi*, *N. kozhantshikovi*, *N. kusnezovi*, *N. maximus* , *N. radobojana*, *N. stavropolicus*, and *Noctuites* sp. from the Chorkraksky and Karagan Horizons, European Russia (Kozlov, 1988); *Noctuites effosus* and *N. haidingeri* from the lignite deposits of Radoboj, Croatia (Heer, 1849); Notodontidae incertae sedis from the Most Formation of the Ùsti Region of the Czech Republic (Prokop, 2003); *Stauropolia nekrutenkoi* from an unspecified horizon in the Stavropol Region, Russia (Skalski, 1988); and an indeterminate noctuoid specimen from the “dysodile beds” of Randecker Maar, Germany (Reiss, 1936).

111. The noctuoid specimen, *Noctuites miocenicus* from an unspecified horizon in the Stavropol Region, Russia (Kozhanchikov, 1957); and *Arctiites deletus* from Tuscany, Italy (Rebel, 1898).

112. Two noctuoid species, *Cerurites wagneri* and *Noctuites gersdorfi* from the lacustrine deposits at Willershausen, Germany (Kernbach, 1967).

113. Sphingid cuticular fragments from the Messel Formation of Germany (Richter and Storch, 1980).

114. Undescribed saturniid cocoons from the Bouxwiller Formation of Alsace, France (Kunz, 2010); and undescribed sphingid specimens in Baltic Amber from the Prussian Formation, Germany (Berendt, 1830).

115. The saturnid species *Rothschildia* (?) *fossilis* from the Florissant Formation of Colorado, USA (Cockerell, 1914).

116. An undescribed larva, probably Sphingidae from the Hiwegi Formation, Lake Victoria region, Kenya (Leakey, 1952); a sphingid larva from the "dysodile beds" of Randecker Maar, Germany (Zeuner, 1927); and the sphingid adult *Mioclanis shanwangiana* from the Shanwang Formation of Shanwang, China (Zhang et al., 1994).

117. The bombycid species, *Bombycites buechii* and *B. oeningensis*, from the "Molasseformatien" of Oeningen, Switzerland (Heer 1849, Kozlov, 1988).

**Literature cited**


Andersen NM, Andersen S: **Kampemyrer og andre danekra fra Limfjordens askeserie–Studiet af en uddød insektfauna fra Danmarks Palaeogen**. *Naturens Verden* 1996, **11/12**:417–432.

Ansorge J: **Revision of the "Trichoptera" described by Geinitz and Handlirsch from the Lower Toarcian of Dobbertin (Germany) based on new material**. *Nova Suppl Entomol* 2002, **15**:55–74.

Bachofen-Echt A: *Der Bernstein und Seine Einschlüsse*. Vienna: Springer-Verlag; 1949.

Berendt GC: *Die Insekten im Bernstein ein Beitrag zur Thiergeschichte der Vorwelt*. Nicolai, Danzig, 38 pp.

Bigot L, Nel A, Nel J: **Description de la première espèce fossile connue de Ptérophore (Lepidoptera, Pterophoridae)**. *Alexanor* 1986, **14**:283−288.

Blair KG: **Insect remains from oil sand in Trinidad**. *Transactions of the Entomological Society of London* 1927, **75**:137–141.

Bonde N, Andersen S, Hald N, Jakobsen SL: *Danekra – Danmarks bedste fossiler*. Copenhagen: Gyldendal; 2008.

Burgeff H: **Die meeralpengrenze der Zygaenen (Lep.), eine mit Hilfe der Populationsanalyse der Arten der Gattung *Zygaena* (Lepidoptera) durchelführte Untersuchungen über die lokalisation und die Beudeutung geographischer Rassen in ihrem Zussammenhang mit der Eiszeit**. *Biologisches Zentralblatt* 1951, **70**:1−23.

Chambers VT: **Burrowing larvae**. *Nature* 1882, **25**: 529.

Cockerell TDA: **Fossil Saturniidae**. *Memoir of the National Academy of Science* 1914, **12**: 171.

Cockerell TDA: **Some American fossil insects**. *Proceedings of the United States National Museum* 1916, **51**: 89–106.

Cockerell TDA: **Two interesting insects in Burmese amber**. *The Entomologist* 1919, **52**:193−195.

Cockerell TDA: **A fossil moth from Florissant, Colorado**. *American Museum Novitates* 1922, **34**:1−2.

Cockerell, TDA: **A new fossil moth from Florissant**. *Psyche* 1926, **33**16−17.

de Serres M: *Géognosie des Terrains Tertiaires ou Tableau des Principaux Animaux Invertébrés des Terrains Marins Tertiaires, du Midi de la France*. Montpellier & Paris: Romathio-Durville; 1829.

Douglas SD, Stockey RA: **Insect fossils in middle Eocene deposits from British Columbia and Washington State: faunal diversity and geological range extensions**. *Canadian Journal of Zoology* 1996, **74**:1140–1157.

Durden CJ, Rose H: **Butterflies from the middle Eocene: the earliest occurrence of fossil Papilionoidea (Lepidoptera)**. *Pearce-Sellards Series, Texas Memorial Museum* 1978, **29**:1−25.

Engel MS, Kinzelbach RK: **A primitive moth from the earliest Eocene Fur Formation ("Mo-clay") of Denmark (Lepidoptera: Micropterigidae)**. *Linzer biologische Beiträge* 2008, **40**:1443−1448.

Evans WP: **Traces of a lepidopterous insect from Middle Waikato Coal-Measures**. *Transactions and Proceedings of the New Zealand Institute* 1931, **62**:99–101.

Fernández-Rubio F, Nel A: ***Neurosymploca*? *oligocenica*, a new fossil species of Lepidoptera Zygaenoidea of the Oligocene of Céreste (Lubéron, France)**. *Boletín Sociedad Entomológica Aragones*a 2000, **27**:7−16.

Fernández-Rubio F, Peñalver E: **Un nuevo ejemplar fósil de *Zygaena*? *turolensis* Fernández-Rubio, Peñalver & Martínez-Delclòs, 1991 (Lepidoptera: Zygaenidae)**. *Estudios del Museo de Ciencias Naturales de Alava* 1994, **9**:39–48.

Fernández-Rubio F, Peñalver E, Martínez-Delclòs X: ***Zygaena*? *turolensis* una nueva especie de Lepidoptera Zygaenidae del Mioceno de Rubielos de Mora (Teruel). Descripción y filogenia**. *Estudios del Museo de Ciencias Naturales de Álava* 1991, **6**:77−93.

Fossil Insect Research Group for Nojiri-ko Excavation: **Fossil insects obtained from the Nojiri-ko Formation during the 10th Nojiri-ko and the 5th Hill Site excavation**. *Monograph of the Association for Geologic Collaboration in Japan* 1990, **37**:93−110.

Fritsch A: **Fossile Arthropoden aus der Steinkohlen- und Kreideformation Böhmens**. *Beiträge zur Paläontologie Österreich-Ungarns* 1882, **2**:1−7, pls. 1−2.

Gall LF, Tiffney BH: **A fossil noctuid moth egg from the Late Cretaceous of eastern North America**. *Science* 1983, **219**:507–509.

Givulescu R: **Pathological elements on fossil leaves from Chiuzbaia (galls, mines and other insect traces)**. *Dãri de Seamã ale Şedinţelor, Institute of Geology and Geophysics* 1984, **18**:123–133.

Gravenhorst H: **Bericht der entomologischen Sektion**. *Schlesische Gesellschaft für Vaterländische Kultur Jahresberichte* 1835, **1834**:88–95.

Grimaldi DA, Engel MS: *Evolution of the Insects*. Cambridge: Cambridge University Press; 2005.

Grimaldi DA, Nascimbene PC: **Raritan (New Jersey) amber**. In *Biodiversity of Fossils in Amber from the Major World Deposits*. Edited by Penny D. Manchester: Siri Scientific Press; 2010: 167–191.

Hall JPW, Robbins RK, Harvey DJ: **Extinction and biogeography in the Caribbean: new evidence from a fossil riodinid butterfly in Dominican amber**. *Proceedings of the Royal Society of London (B)* 2004, **271**:797−801.

Harris AC, Raine JI: **A sclerite from a late Cretaceous moth (Insecta: Lepidoptera) from Raikaia Gorge, Canterbury, New Zealand**. *Journal of the Royal Society of New Zealand* 2002, **32**:457–462.

Heer O: *Die Insektenfauna der Tertiärgebilde von Oeningen und von Radoboj in Croatien. Volume 2*. Leipzig: Wilhelm Engelmann; 1849.

Heer O: **Ueber die fossilen Insekten von Aix in der Provence**. *Vierteljahrsschrift der Naturforschenden Gesellschaft in Zürich* 1856, **1**:1−40.

Heer O: *Recherches sur le Climat et la Végétation du Pays Tertiaire*. Geneva & Paris: Winterthur; 1861.

Heyden C: **Gliederthiere aus der Braunkohle des Niederrhein's, der Wetterau und der Röhn**. *Palaeontographica* 1862, **10**:62−82.

Hickey LJ, Hodges RW: **Lepidopteran leaf mine from the early Eocene Wind River Formation of northeastern Wyoming**. *Science* 1975, **189**:718–720.

Hurd PDJ, Smith RF, Durham JW: **The fossiliferous amber of Chiapas, Mexico**. *Ciencia* 1962, **21**:108–118.

Jarzembowski EA: **Report of Easter Field Meeting: the Lower Tertiaries of the Isle of Wight, 27–31.III.1975**. *Tertiary Research* 1976, **1**: 11–16.

Jarzembowski EA: **Fossil insects from the Bembridge Marls, Palaeogene of the Isle of Wight, southern England**. *Bulletin of the British Museum of Natural History (Geology)* 1980, **33**:237–293.

Jarzembowski EA: **A century plus of fossil insects**. *Proceedings of the Geologists' Association* 1989, **100**:433−449.

Jarzembowski EA: **Checklist of Tertiary insects from Dorset**. *Proceedings of the Dorset Natural History and Archaeological Society* 1995, **116**:145–146.

Joseph NC: **Eocene flora and fauna unearthed at Republic**. *Washington Geologic Newsletter* 1986, **14**:3–5.

Kernbach K: **Über die bischer im Pliozän von Willershausen gefundenen Schmetterlings- und Raupenreste**. *Bericht der Naturhistorischen Gesellschaft zu Hannover* 1967, **111**:103−108.

Klebs R: **Ueber die Fauna des Bernsteins**. *Versammlung Deutscher Naturforscher und Ärzte in Heidelberg* 1890, **18**:268–271.

Knowlton FH: **A fossil flora from the Frontier Formation of southwestern Wyoming**. *United States Geological Survey Professional Paper* 1917, **108**:73–95, pls. 1–39.

Koponen M, Nuorteva M: **Über subfossile Waldinsekten aus dem Moor Piionsuo in Südfinnland**. *Acta Entomologica Fennica* 1973, **29**:1−84.

Kozhanchikov IV: **Novy predstavitel' sem Cossidae iz Miotsenovikh otlozheny Kavkaza (Lepidoptera, Insecta)**. *Doklady Akademiya Nauk SSSR* 1957, **113**:675–677.

Kozlov MV: **Noviye Molevidniye Cheshuyekriliye iz Baltiiskogo Yantara**. *Paleontologicheskiy Zhurnal* 1987, **1987**:59–67.

Kozlov MV: **Paleontology of the Lepidoptera and problems of phylogeny of the order Papilionoidea**. In *The Cretaceous Biocoenotic Crisis and the Evolution of Insects*. Edited by Ponomarenko AG. Moscow: Nauka Press; 1988:16−69.

Kozlov MV: **New Lepidoptera (Papilionoidea) from the Upper Jurassic and Lower Cretaceous**. *Paleontologicheskiy Zhurnal* 1989, **4**:37−42.

Kozlov MV, Ivanov VD, Rasnitsyn AP: **Order Lepidoptera Linné, 1758. The butterflies and moths (= Papilionida Laicharting, 1781)**. In *History of Insects*. Edited by Rasnitsyn AP, Quicke DLJ. Dordrecht, Boston & London: Kluwer Academic Publishers; 2002:220–227.

Krassilov VA: **Mines and galls on fossil leaves from the Late Cretaceous of southern Negev, Israel**. *African Invertebrates* 2007, **48**:13–22.

Krassilov V, Shuklina S: **Arthropod trace diversity on fossil leaves from the mid-Cretaceous of Negev, Israel**. *Alavesia* 2008, **2**:239–245.

Kristensen NP, Skalski AW: **Phylogeny and palaeontology**. In *Handbook of Zoology. Volume IV Arthropoda: Insecta. Part 35 Lepidoptera, Moths and Butterflies 1: Evolution, Systematics, and Biogeography*. Edited by Kristensen NP. Berlin & New York: Walter de Gruyter; 1998:7–25.

Kühne WG, Kubig L, Schlüter T: **Eine Micropterygide (Lepidoptera, Homoneura) aus mittelcretazischem Harz Westfrankreichs**. *Mitteilungen der Deutschen Entomologischen Gesellschaft* 1973, **32**:61–64.

Kunz P: **30 ans déjà**. *ASAM Bulletin* 2010, **2010**:39–45.

Kupryjanowicz J: **The Amber Treasure Trove, Part I. Arthropods in Baltic amber and their photographic record**. *Museum of the Earth Documentary Studies* 2001, **18**:19–59.

Kurz MA, Kurz ME: Taxonomy Online: *Micropterix gertraudae* Kurz M. A. & M. E. Kurz, 2010. [http://www.nkis.info/nkis/extaustaxonshow.cgi?uid=guest&tax=161783&lang=e/]

Kusnezov N: ***Oligamatites martynovi*, gen. et sp. n., a fossil amatid lepidopteron from the Oligocene beds of Central Asia**. *Doklady Akademiya Nauk SSSR* 1928, **20**: 431–436.

Kusnezov N: *A Revision of Amber Lepidoptera*. Moscow & Leningrad: Paleontological Institute, Academy of Sciences; 1941.

Labandeira CC: **Plant-insect associations from the fossil record**. *Geotimes* 1998, **43**:18–24.

Labandeira CC**: Paleobiology of middle Eocene plant-insect associations from the Pacific Northwest: A preliminary report**. *Rocky Mountain Geology* 2002a, **37**:31–59.

Labandeira CC: **The history of associations between plants and animals**. In *Plant–Animal Interactions: An Evolutionary Approach*. Edited by Herrera CM, Pellmyr O. London: Blackwell Science; 2002b:28–74, 248–261.

Labandeira CC: **Assessing the fossil record of plant–insect associations: ichnodata versus body-fossil data**. In *Sediment–Organism Interactions: A Multifaceted Ichnology*. Edited by Bromley RG, Buatois LA, Mángano F, Genise JF, Melchor RN. Tulsa: Society for Sedimentary Geology; 2007:9–26.

Labandeira CC, Dilcher DL, Davis DR, Wagner DL: **Ninety-seven million years of angiosperm-insect association: Paleobiological insights into the meaning of coevolution**. *Proc Natl Acad Sci USA* 1994, **91**:12278–12282.

Labandeira CC, Johnson KR, Lang PJ: **Preliminary assessment of insect herbivory across the Cretaceous–Tertiary boundary: Major extinction and minimum rebound**. *Geological Society of America Special Paper*, 2002a, **361**:297–327.

Labandeira CC, Johnson KR, Wilf P: **Impact of the terminal Cretaceous event on plant-insect associations**. *Proceedings of the National Academy of Sciences, USA* 2002b, **99**:2061–2066.

Larsson SG: **Paleobiology and mode of burial of the insects of the Lower Eocene Mo-clay of Denmark**. *Bulletin of the Geological Society of Denmark* 1975, **24**:193–209.

Leakey LSB: **Lower Miocene invertebrates from Kenya**. *Nature* 1952, **169**:624–625.

Leestmans R: **Les lépidoptères fossiles trouvés en France (Insecta Lepidoptera)**. *Linneana Belgica*, 1983, **9**:64–89.

Lewis SE: **Lepidopterous feeding damage of live oak leaf (*Quercus convexa* Lesquereux) from the Ruby River Basin (Oligocene) of southwestern Montana**. *Journal of Paleontology*, 1976, **50**:345–346.

Lewis SE: **Miocene insects from the Clarkia deposits of northern Idaho**. In *Late Cenozoic History of the Pacific Northwest*. Edited by Smiley CJ. Oregon: American Association for the Advancement of Science, Pacific Division; 1985: 245–264.

Lewis SE: **Insects of the Klondike Mountain Formation, Republic, Washington**. *Washington Geology* 1992, **20**:15–19.

Liebhold AM, Voney WJA, Schorn HE: **An unidentified leaf mine in fossil *Mahonia reticulata* (Berberidaceae)**. *The Canadian Entomologist* 1982, **114**:455–456.

McNamara M, Briggs DEG, Orr PJ, Wedmann S, Noh H, Cao H: **Fossilized biophotonic nanostructures reveal the original colors of 47-million-year-old moths**. *PLoS Biology* 2011, **9**:e1001200.

Martínez-Delclós X, Arillo A, Ortuño V, Peñalver E: **El ámbar del Cretácico Inferior de Peñacerrada (Álava, España)**. *Temas Geológico-Mineros ITGE* 1999, **26**: 13–17.

Martins-Neto RG: **Novos insetos Terciários do Estado de São Paulo**. *Revista Brasileira de Geociências*, 1989, **19**:375–386.

Martins-Neto RF: **A paleontomofauna da Formação Tremembé (Bacia de Taubaté), Oligoceno do Estado de São Paulo: descrição de novos lepidópteros (Insecta)**. *Acta Geologica Leopoldensia* 1998a, **41**:75−82.

Martins-Neto RF: **A paleontomofauna da Formação Tremembé (Bacia de Taubaté), Oligoceno do Estado de São Paulo: novos Hemiptera, Auchenorrhyncha, Hymenoptera, Coleoptera e Lepidoptera (Insecta)**. *Revista Universidade Guarulhos, Geosciências* 1998b, **3**:58−70.

Martins-Neto RF, Kucera-Santos JC, de Moraes Vieira FR, de Campos-Fragosa LM: **Nova espécie de borboleta (Lepidoptera: Nymphalidae: Satyrinae [sic] da Formação Tremembé, Oligoceno do Estado de São Paulo**. *Acta Geologica Leopoldensia* 1993, **16**:5−16.

Martins-Neto RF, Vulcano MA: **Amphiesmenoptera (Trichoptera + Lepidoptera) na Formação Santana (Cretáceo Inferior) Bacia do Araripe, Nordeste do Brasil. I. Lepidoptera (Insecta)**. *Anais da Academia Brasileira de Ciências* 1989, **61**:459−466.

Menge A: *Lebenszeichen Vorweltlicher, im Bernstein Eingeschlossener Thiere*. Danzig: A.W. Kafemann; 1856.

Mérit X, Mérit M: ***Problongos baudiliensis* genus novus & species nova: un nouveau Lépidoptère fossile découvert dans la diatomite du Miocène Supérieur de Saint-Bauzile (Ardèche, F-07) (Lepidoptera, Geometridae, Ennominae)**. *Revue dans Lépidoptéristes de France* 2008, **17**:29−33.

Mey W: **On the systematic position of *Baltimartyria* Skalski, 1995, and description of a new species from Baltic amber (Lepidoptera, Micropterigidae)**. *ZooKeys* 2011, **130**:331−342.

Meyer HW: *The Fossils of Florissant*. Washington DC & London: Smithsonian Books; 2003.

Nekrutenko YP: **A new Tertiary nymphalid (Lepidoptera, Rhopalocera)**. *Palentologicheskiy Zhurnal* 1965, **4**:97−99.

Nel A, Descimon H: **Une nouveau espèce de Lépidoptère fossile du Stampien de Céreste (Lepidoptera−Satyrinae)**. *Géologie Méditerranèene*, 1994, **11**:287−293.

Nel A, Nel J: **A propos de deux Lépidoptère fossile du Stampien d' Aix-en-Provence (Lepidoptera−Hesperidae et Nymphalidae Satyrinae)**. *Bulletin du Muséum National d'Histoire Naturelle, Paris* (4) 1986, **8**:343−350.

Nel A, Nel J, Balme C: **Un noveau lépidoptère Satyrinae fossile de l'Oligocene du sud-est de la France (Insecta, Lepidoptera, Nymphalidae)**. *Linneana Belgica*, 1993, **14**:20−36.

Nuorteva M, Kinnunen KA: **Insect frass in Baltic amber**. *Bulletin of the Geological Society of Finland* 2008, **80**:105–124.

Opler PA: **Fossil lepidopterous leaf mines demonstrate the age of some insect-plant relationships**. *Science* 1973, **179**:1321–1323.

Opler PA: **Fossil leaf-mines of *Bucculatrix* (Lyonetiidae) on *Zelkova* (Ulmaceae) from Florissant, Colorado**. *Journal of the Lepidopterists' Society* 1982, **36**:145–147.

Oustalet ME: **Recherches sur les insectes fossiles des Terrains Tertiares de la France**. *Annales des Sciences Géologiques* 1870, **2**:1−175.

Peñalver E, Delclós X: **Insectos del Mioceno inferior de Ribesalbes (Castellón, España). Interacciones planta-insecto**. *Treballs del Museu de Geologia de Barcelona* 2004, **12**:69–95.

Peñalver E, Engel MS: **Two wasp families rare in the fossil record (Hymenoptera): Perilampidae and Megaspilidae from the Miocene of Spain**. *American Museum Novitates* 2006, **3540**:1–12.

Peñalver E, Grimaldi DA: **New data on Miocene butterflies in Dominican amber (Lepidoptera: Riodinidae and Nymphalidae) with the description of a new nymphalid**. *American Museum Novitates* 2006, **3519**:1–17.

Piton L: **Addition a la faune entomologique des cinerites du La Chambon (Puy-de -Dome)**. *Revue Scientifique du Bourbonnais et du Centre de la France* 1936, **1936**:16–24.

Piton L: *Paléontologie du Gisement Éocene de Menat (Puy-de-Dome) (Flore et Faune)*. Clermont-Ferrand: Imprimeries Paul Vallier; 1940.

Poinar Jr GO: *Life in Amber*. Stanford: Stanford University Press; 1992.

Poinar Jr GO, Brown AE: ***Hymenaea mexicana* sp. nov. (Leguminosae: Caesalpinioideae) from Mexican amber indicates Old World connections**. *Botanical Journal of the Linnean Society* 2002, **139**:125–132.

Poinar Jr GO, Brown JW: **A new fossil tortricid (Lepidoptera: Tortricidae) from Dominican amber**. *Entomologica Scandinavica* 1993, **23**:25−29.

Prokop J: **Remarks on palaeoenvironmental changes based on reviewed Tertiary insect associations from the Krušné hory piedmont basins and the České středhoří Mts in northwestern Bohemia (Czech Republic)**. *Acta Zoological Cracoviensia* 2003, **46** (Suppl):329–344.

Rasnitsyn AP, Ross AJ: **A preliminary list of arthropod families present in the Burmese amber collection at the Natural History Museum, London**. *Bulletin of the Natural History Museum, London (Geology)* 2000, **56**:21–24.

Rasnitsyn AP, Zherikhin VV: **Appendix: Alphabetic list of selected insect fossil sites. Impression fossils**. In *History of Insects*. Edited by Rasnitsyn AP, Quicke DLJ. Dordrecht: Kluwer Academic Publishers; 2002: 437−444.

Rebel H: **Fossil Lepidopteren aus der Miocänformation von Gabbro**. *Sitzungsberichte der kaiserlichen Akademie der Wissenschaften*, 1898, **107**:731−745.

Rebel H: **Bernstein−Lepidopteren (aus der Sammlung Bachofen-Echt)**. *Paleobiologica* 1934, **6**:1−6, pl. 1.

Rebel H: **Mikrolepidopteren aus dem baltischen Bernstein**. *Die Naturwissenschaften* 1936, **24**:519−520.

Reiss H: **Ein Zygaenenfund aus der Tertiärzeit**. *Entomologische Rundschau* 1936, **53**:554−556.

Richter G, Storch G: **Beiträge zur Ernährungsbiologie eozäner Fledermäuse aus der "Grube Messel"**. *Natur und Museum* 1980, **110**:353–367.

Ross AJ, Mellish C, York P, Crighton B: **Burmese amber**. In *Biodiversity of Fossils in Amber from the Major World Deposits*. Edited by Penny D. Manchester: Siri Scientific Press; 2010: 208–235.

Rozefelds AC: **Lepidoptera mines in *Pachypteris* leaves (Corystospermaceae: Pteridospermophyta) from the Upper Jurassic/Lower Cretaceous Battle Camp Formation, north Queensland**. *Proceedings of the Royal Society of Queensland* 1988, **99**:77–81.

Scudder SH: **Results of an examination of a small collection of fossil insects obtained by Prof. Wm. Denton in the Tertiary beds of Green River, Colorado**. *Proceedings of the Boston Society of Natural History* 1867, **11**:117–118.

Scudder SH: **Fossil butterflies**. *Memoirs of the American Association for the Advancement of Science* 1875, **1**:1−99.

Scudder SH: **An account of some insects of unusual interest from the Tertiary rocks of Colorado and Wyoming**. *Bulletin of the United States Geological Survey of the Territories* 1878, **4**: 519−543.

Skalski AW: **Studies on the Lepidoptera from fossil resins. Part VI. *Tortricidrosis inclusa* gen. et spec. nov. from the Baltic amber (Lep., Tortricidae)**. *Deutsche Entomologische Zeitschrift, N. F.* 1973a, **20**:339–344.

Skalski AW: **Studies on the Lepidoptera from fossil resins. Part II. *Epiborkhausenites obscurotrimaculatus* gen. et sp. nov. (Oecophoridae) and a tineid-moth discovered in the Baltic amber**. *Acta Palaeontologica Polonica* 1973b, **28**:153−160.

Skalski AW: **Zwei neue Gattungen und Arten der Familie Tineidae aus dem Baltischen Bernstein**. *Beiträge zur Entomologie* 1974, **24**:97−104.

Skalski AW: **Les lépidoptères fossiles de l'ambre, Etat actuel de nos connaissances (3^me^ partie et fin)**. *Linneana Belgica* 1976a, **6**:221–233.

Skalski AW: **Les lépidoptères fossiles de l'ambre, Etat actuel de nos connaissances (2^me^ partie)**. *Linneana Belgica*, 1976b, **6**:195–208.

Skalski AW: **Studies on the Lepidoptera from fossil resins. Part 1. General remarks and descriptivos of new genera and especies of the families Tineidae and Oecophoridae from the Baltic amber**. *Prace Muzeum Ziemi* 1977, **26**: 3−24.

Skalski AW: **Records of oldest Lepidoptera**. *Nota Lepidopterologica* 1979a, **2**: 61–66.

Skalski AW: **Study on the lepidoptera from fossil resins. Part II. *Palaeodepressaria hannemanni* gen. et sp. nov. (Oecophoridae) from the Baltic amber**. *Prace Muzeum Ziemi* 1979b, **32**:101−107.

Skalski AW: **Motyle (Lepidoptera) w bursztynie baltyckim**. *Wiadomosci Entomologiczne* 1985, **6**:207–210.

Skalski AW: ***Stauropolia nekrutenkoi* gen. et. sp. n. (Lepidoptera, Arctiidae)–a new fossil Tiger-moth from Miocene of the Caucasus**. *Vestnik Zoologii* 1988, **1988**:21–25.

Skalski AW: **An annotated review of all fossil records of Lower Lepidoptera**. *Bulletin of the Sugadaira Montane Research Center* 1990a, **11**:125–128.

Skalski AW: **Lepidoptera in fossil resins with emphasis on new investigations**. *Prace Muzeum Ziemi* 1990b, **41**:163–164.

Skalski AW: **Studies on the Lepidoptera from fossil resins. Part III. Two new genera and species of Tortricidae from the Baltic Amber**. *Annals of the Upper Silesian Museum (Entomology)* 1992, **3**:137−146.

Skalski AW, Veggiani A: **Fossil resin in Sicily and the northern Apennines: geology and organic content**. *Prace Muzeum Ziemi* 1990, **41**:37−49.

Sobczyk T, Kobbert MJ: **Die Psychidae des baltischen Bernsteins**. *Nota lepidopterologica* 2009, **32**:13–22.

Sohn J-C, Labandeira C, Davis D, Mitter C: **An annotated catalog of fossil and subfossil Lepidoptera (Insecta: Holometabola) of the world**. *Zootaxa* 2012, **3286**:1−132.

Stephenson J: **Evidence of Plant/Insect Interactions in the Late Cretaceous and Early Tertiary**. *PhD Thesis*. University of London, Department of Geology; 1991.

Straus A: **Eine Gangmine im Eozän von Messel**. *Aufschluß* 1976, **27**:445−446.

Straus A: **Gallen, Minen und andere Fraßspuren im Pliozän von Willershausen am Harz**. *Verhandlungen des Botanischen Vereins der Provinz Brandenburg* 1977, **113**:43–80.

Théobald N: *Les Insectes Fossiles des Terrains Oligocenes de France*. Nancy: Imprimerie Georges Thomas; 1937.

Tindale NB: **A butterfly-moth (Lepidoptera, Castniidae) from the Oligocene shales of Florissant, Colorado**. *Journal of Research on the Lepidoptera* 1985, **24**:31−40.

Whalley PES: **New taxa of fossil and recent Micropterigidae with a discussion of their evolution and a comment on the evolution of Lepidoptera (Insecta)**. *Annals of the Transvaal Museum* 1978, **31**:71–86.

Whalley PES: **The systematics and biogeography of the Lower Jurassic insects of Dorset, England**. *Bulletin of the British Museum of Natural History (Geology)* 1985, **39**:107–189.

Wilf P, Labandeira CC, Johnson KR, Cúneo NR: **Richness of plant-insect associations in Eocene Patagonia: A legacy for South American biodiversity**. *Proceedings of the National Academy of Sciences, USA* 2005, **102**:8944–8948.

Zeuner FE: **Eine Sphingidenraupe aus dem Obermiozän von Böttingen**. *Palaeontologische Zeitschrift*, 1927, **8**:321–326.

Zeuner FE: **Two new fossil butterflies of the family Pieridae**. *Annals and Magazine of Natural History*, 1942, **10**:140−144.

Zhang J: *Fossil Insects from Shanwang, Shandong, China*. Jinan: Shandong Science Technology Publishing House; 1989.

Zhang J, Sun B, Zhang X: *Miocene Insects and Spiders from Shanwang*. Beijing: Science Press; 1994.

Zherikhin VV, Sukacheva ID: **On Cretaceous "ambers" (retinites) with insects of North Siberia**. *Doklady na XXIV Ezhegodnom Tshtenii Pamiati N. A. Cholodkovskogo* 1973, **1971**:3–48.
